# Structural insights into TRPV2 activation by small molecules

**DOI:** 10.1038/s41467-022-30083-3

**Published:** 2022-04-28

**Authors:** Ruth A. Pumroy, Anna D. Protopopova, Tabea C. Fricke, Iris U. Lange, Ferdinand M. Haug, Phuong T. Nguyen, Pamela N. Gallo, Bárbara B. Sousa, Gonçalo J. L. Bernardes, Vladimir Yarov-Yarovoy, Andreas Leffler, Vera Y. Moiseenkova-Bell

**Affiliations:** 1 Department of Systems Pharmacology and Translational Therapeutics, Perelman School of Medicine, University of Pennsylvania, Philadelphia, PA 19104 United States; 2 Department of Anesthesiology and Intensive Care Medicine, Hannover Medical School, Hannover, 30625 Germany; 3 Department of Physiology and Membrane Biology, University of California, Davis, CA 95616 United States; 4 Instituto de Medicina Molecular, Faculdade de Medicina, Universidade de Lisboa, Lisboa, 1649-028 Portugal; 5 Department of Chemistry, University of Cambridge, Lensfield Road, Cambridge, CB2 1EW UK

**Keywords:** Cryoelectron microscopy, Transient receptor potential channels

## Abstract

Transient receptor potential vanilloid 2 (TRPV2) is involved in many critical physiological and pathophysiological processes, making it a promising drug target. Here we present cryo-electron microscopy (cryo-EM) structures of rat TRPV2 in lipid nanodiscs activated by 2-aminoethoxydiphenyl borate (2-APB) and propose a TRPV2-specific 2-ABP binding site at the interface of S5 of one monomer and the S4-S5 linker of the adjacent monomer. In silico docking and electrophysiological studies confirm the key role of His521 and Arg539 in 2-APB activation of TRPV2. Additionally, electrophysiological experiments show that the combination of 2-APB and cannabidiol has a synergetic effect on TRPV2 activation, and cryo-EM structures demonstrate that both drugs were able to bind simultaneously. Together, our cryo-EM structures represent multiple functional states of the channel, providing a native picture of TRPV2 activation by small molecules and a structural framework for the development of TRPV2-specific activators.

## Abstract (web summary)

A non-selective calcium channel transient receptor potential vanilloid 2 (TRPV2) is a potential drug target. Here, the authors employ cryo-electron microscopy, in silico docking, and electrophysiology to identify a binding site for an activator 2-aminoethoxydiphenyl borate (2-APB) in this channel.

## Introduction

Transient receptor potential vanilloid 2 (TRPV2) is a Ca^2+^-permeable non-selective cation channel belonging to the vanilloid subfamily of the transient receptor potential (TRP) channels^1,2^. TRPV2 is expressed in most organs of the human body, including the heart, white blood cells, the pancreas, the central and peripheral nervous systems, the bladder, the prostate, and the placenta^3^. TRPV2 has been shown to play a significant role in maintaining physiological cardiomyocyte function, with a potential therapeutic use of TRPV2 blockade in cardiomyopathy^4–6^. TRPV2 plays a role in phagocytosis in macrophages^7,8^, placental development^9^, T-cell activation^10,11^, and insulin secretion in pancreatic β-cells^12,13^. Beyond its routine functions in healthy cells, TRPV2 has been shown to play a significant role in the progression, drug resistance and metastasis of different forms of cancer^14^. Thus, TRPV2’s role in both healthy and malignant tissues makes it a promising target for drug discovery and the development of novel therapeutics in cardiovascular diseases, cancer, and additional pathophysiological conditions.

A key limiting factor in understanding precise TRPV2 molecular function is the lack of specific modulators of the channel. While endogenous modulation of TRPV2 remains largely unexplored, there are several known non-specific exogenous activators of TRPV2, including 2-aminoethoxydiphenyl borate (2-APB), cannabidiol (CBD) and probenecid, and exogenous inhibitors, including ruthenium red, trivalent cations, tranilast, and piperlongumine^2,15^. To aid the rational development of modulators with increased efficacy and TRPV2 specificity, more structural information is needed to determine binding sites for the known ligands and track channel gating.

Early structural studies of TRPV2 in the absence of TRPV2 modulators hypothesized mechanisms of channel gating^16–20^, but due to modifications in the protein sequence and the use of detergents those structures did not provide a comprehensive picture of wildtype TRPV2 gating in a native lipid environment. Recently, our lab reported CBD-bound TRPV2 structures in lipid nanodiscs revealing CBD binding to a therein reported ligand binding site^21^. Nevertheless, the CBD-bound TRPV2 structures did not reveal a mechanism for TRPV2 opening by small molecules^21^. Thus, it is essential to study how other known TRPV2 exogenous activators interact and gate the channel in order to get a full picture of the gating cycle and discover potential ligand-binding sites.

To address the need for structural information of TRPV2 ligand-binding and gating, here we use cryo-electron microscopy (cryo-EM) to determine the structures of wildtype rat TRPV2 in the presence of 2-APB in lipid nanodiscs, which reveals a binding pocket for 2-APB between the S5 helix and the S4-S5 linker of two adjacent monomers. In silico docking and electrophysiological data validate the proposed binding site for 2-APB and confirm a key role for His521 and Arg539 in the binding pocket. Additionally, electrophysiological data reveal that CBD acts as a potentiator for 2-APB activation of TRPV2 and cryo-EM structures confirm that both drugs can bind simultaneously at their unique binding pockets. Cryo-EM also allow us to resolve two distinct conformations of TRPV2 bound to 2-APB: an activated state and an inactivated state, leading us to propose a wedge mechanism for TRPV2 modulation by 2-APB. While 2-APB does not bind in the vanilloid pocket, it induces conformational changes in TRPV2 that lead to the expulsion of the vanilloid lipid as seen in other TRPV channels upon activation. Together, our results put TRPV2 in line with activation mechanisms established for other TRPV channels and lay the foundation for future development of TRPV2-specific modulators.

## Results

### Activated and inactivated structures of 2-APB-bound TRPV2

2-APB is a well-established activator of the TRPV1-3 channels^22^. Recent structural studies^23–25^ revealed a consensus 2-APB binding site for TRPV3 positioned between the Pre-S1 helix and the TRP helix, which agrees with previous functional data^26^. Since the critical histidine residue of the TRPV3 2-APB binding site is not conserved in TRPV2^26^ (Supplementary Fig. 1A), we hypothesized that TRPV2 interacts with 2-APB through a different binding site that could be used to develop TRPV2-specific therapeutics and unravel the TRPV2 gating mechanism.

In order to obtain an activated structure of the TRPV2 channel and identify the TRPV2 2-APB binding site, we collected a large cryo-EM dataset of rat TRPV2 reconstituted in lipid nanodiscs and incubated with 1 mM 2-APB for 5 min before preparing the grids. The dataset showed extensive heterogeneity and was analyzed using the 3D variability tool^27^ from cryoSPARC^28^ (Supplementary Fig. 2). This generated continuous families of structures reflecting the major movements that appear in the sample and assigned each particle to a specific position within each family. The family of states for the C4 mode of movement for 2-APB-bound TRPV2 showed substantial conformational changes and partial opening of the channel (Supplementary Movie 1). The particles corresponding to the most open and the most closed states were isolated and refined independently, providing two distinct structures with C4 symmetry which we named TRPV2 2-APB-bound “activated” state (TRPV2_2APB_AC_) at 4.15 Å resolution and TRPV2 2-APB-bound “inactivated” state (TRPV2_2APB_IAC_) at 3.5 Å resolution (Supplementary Fig. 3, Table 1).

**Table 1:** Cryo-EM data collection and model statistics.

	TRPV2_2APB_AC_ (EMD-24110, PDB 7N0N)	TRPV2_2APB_IAC_ (EMD-24109, PDB 7N0M)	TRPV2_2APB_CBD_AC_ (EMD-25650, PDB 7T37)	TRPV2_2APB_CBD_IAC_ (EMD-25651, PDB 7T38)
Data collection and processing				
Magnification	81,000×	81,000×	81,000×	81,000×
Voltage (kV)	300	300	300	300
Defocus range (μm)	−0.8 to −3.0	−0.8 to −3.0	−0.8 to −3.0	−0.8 to −3.0
Pixel size (Å)	1.07	1.07	1.07	1.07
Particles from 2D classification (no.)	1,857,876	1,857,876	820,578	820,578
Final particles (no.)	8258	92,948	30,563	33,404
Symmetry	C4	C4	C4	C4
Map sharpening B factor (Å^2^)	−145	−155	−120	−130
Map resolution (Å)	4.15	3.5	3.7	3.8
FSC threshold	0.143	0.143	0.143	0.143
Model Refinement				
Model resolution (FSC 0.143) (Å)	4.15	3.5	3.7	3.8
Model composition				
Nonhydrogen atoms	19,700	19,780	20,176	19,404
Protein residues	2468	2476	2476	2432
Ligands		FZ4: 4	FZ4: 4	
			P0T: 4	
R.M.S. deviations				
Bond lengths (Å)	0.006	0.009	0.006	0.004
Bond angles (°)	1.014	1.051	0.840	0.784
Validation				
MolProbity score	1.48	1.67	1.47	1.38
Clashscore	4.68	5.53	4.33	4.09
Rotamers outliers (%)	0.38	0.57	0	0.39
CαBLAM outliers (%)	1.26	2.47	1.68	2.38
EMRinger Score	1.31	2.63	2.15	1.54
Ramachandran plot				
Favored (%)	96.38	94.58	96.17	96.86
Allowed (%)	3.62	5.42	3.83	3.14
Disallowed (%)	0.00	0.00	0.00	0.00

**Fig. 1:**
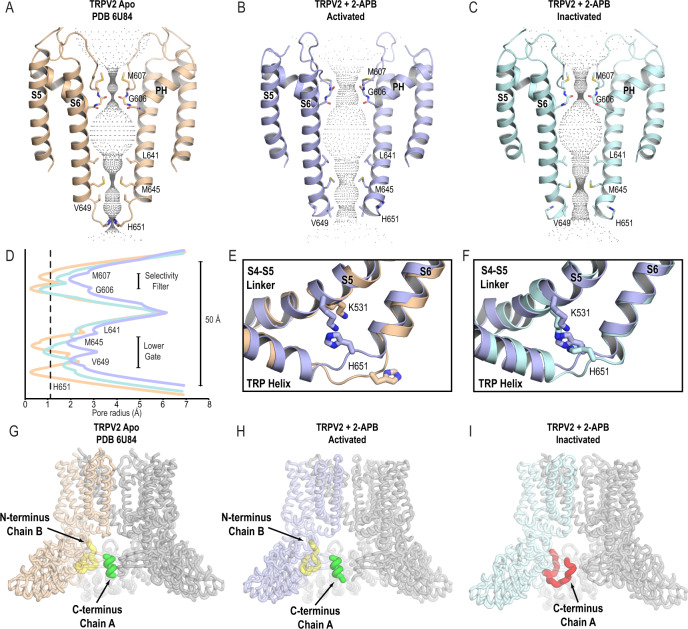
2-APB-bound TRPV2 in activated and inactivated states. HOLE-generated pore profiles of TRPV2_Apo1_ (PDB 6U84) (**A**), TRPV2_2APB_AC_ (**B**), and TRPV2_2APB_IAC_ (**C**). **D** Graphical representation of the pore profiles of TRPV2_Apo1_ (wheat), TRPV2_2APB_AC_ (light blue), TRPV2_2APB_IAC_ (light cyan). The radius of a dehydrated calcium ion is marked by a dotted gray line at 1.1 Å. **E**, **F** His651 is inserted into the pore in the apo state (TRPV2_Apo1_, wheat), but rotates out of the central channel of the pore on channel activation (TRPV2_2APB_AC_, light blue) to form a cation–π bond with Lys531. During channel inactivation (TRPV2_2APB_IAC_, light cyan), His651 remains stably rotated out of the pore. TRPV2_Apo1_ (**G**) and TRPV2_2APB_AC_ (**H**) feature the active conformation of the cytoplasmic regulatory switch, with the C-terminus forming a helix (green) inside the ankyrin repeat domain (ARD) skirt and the N-terminus (yellow) forming a loop at the multimerization interface between the β-sheet region and an ARD. TRPV2_2APB_IAC_ (**I**) features the inactive conformation of the cytoplasmic regulatory switch, with the C-terminus (red) forming a loop that wraps around the β-sheet region.

We compared TRPV2_2APB_AC_ and TRPV2_2APB_IAC_ with each other and with the previously identified fully closed apo state of the channel in nanodiscs (TRPV2_Apo1_, PDB 6U84)^21^ and all three structures were remarkably different at their pore profiles and at the cytoplasmic regulatory switch^23^ (Fig. 1A–D, G–I). The pore of TRPV2_2APB_AC_ was partially open (Fig. 1B) with a pore radius of 2.0 Å at Gly606 in the selectivity filter and 1.9 Å at the Met645 in the lower gate (Fig. 1D). Thus, the diameter of the TRPV2_2APB_AC_ pore could allow a partially dehydrated Ca^2+^ ion to go through. The S6 helix of TRPV2_2APB_AC_ remained entirely α-helical similar to TRPV2_Apo1_ (Fig. 1A, B), but with a significant conformational change at the bottom of S6 where the sidechain of His651 was rotated out of the pore by 180°. In this new position, His651 stabilized TRPV2_2APB_AC_ in the partially open conformation through the newly formed cation–π interaction between His651 and Lys531 from the S4-S5 linker (Fig. 1B, E). In contrast, in TRPV2_Apo1_ His651 tightly seals the lower gate (Fig. 1A, E). At the cytoplasmic regulatory switch, TRPV2_2APB_AC_ was similar to TRPV2_Apo1_ (Fig. 1G) and characterized by the presence of a stable C-terminal helix (residues 710-720, Fig. 1H) and N-terminal loop (residues 30–45, Fig. 1H), suggesting that we captured TRPV2 in an activated state that is an intermediate or partially open.

The TRPV2_2APB_IAC_ state was closed at both lower and upper gates (Fig. 1C, D). The narrowest constrictions in the pore were at Gly606 in the selectivity filter and at the Met645 in the lower gate, where the pore radius was 0.6 Å and 0.9 Å, respectively (Fig. 1C, D). The S6 helix of TRPV2_2APB_IAC_ was entirely α-helical (Fig. 1C) and the His651 at the bottom of S6 was still rotated out of the pore, forming a cation-π interaction with Lys531 (Fig. 1F). At the cytoplasmic regulatory switch, TRPV2_2APB_IAC_ had a C-terminal loop (residues 710-726, Fig. 1I) which displaced the N-terminal loop. Therefore, we interpreted TRPV2_2APB_IAC_ as an inactivated state of TRPV2.

### Identification and validation of the 2-APB binding site

**Fig. 2:**
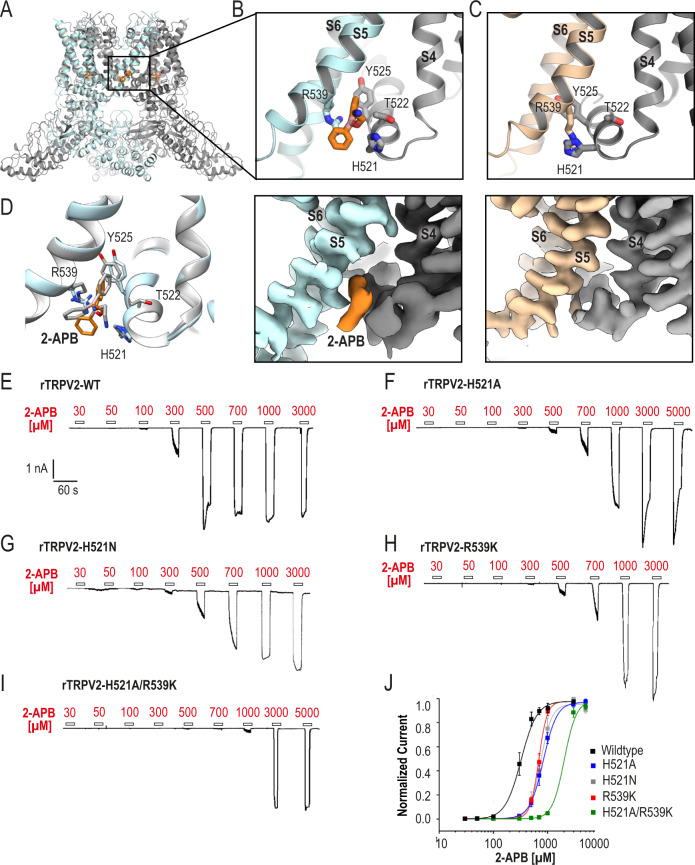
Identification and validation of 2-APB binding site. **A** Cartoon representation of TRPV2_2APB_IAC_. Cartoon representation (upper panel) and map density (lower panel) of the 2-APB binding site in TRPV2_2APB_IAC_ (**B**) and TRPV2_Apo1_ (PDB 6U84) (**C**). One monomer is colored light cyan or wheat, the adjacent monomer is colored gray; 2-APB is colored orange. Maps contoured at *σ* = 5. **D** Structural comparison of the top 2-APB docking model (gray) and TRPV2_2APB_IAC_ (light cyan and orange). Representative whole-cell patch clamp current traces displaying a concentration-dependent activation of wildtype rat TRPV2 (**E**), His521Ala rat TRPV2 (**F**), His521Asn (**G**), Arg539Lys rat TRPV2 (**H**), and His521Ala/Arg539Lys rat TRPV2 (**I**) by 2-APB. Cells were held at −60 mV and increasing concentrations of 2-APB were applied. **J** Dose–response curves for 2-APB-evoked activation of wildtype rat TRPV2 (black, EC_50_ = 322 ± 27 μM *n* = 8), Arg539Lys rat TRPV2 (red, EC_50_ = 683 ± 78 μM, *n* = 4), His521Ala rat TRPV2 (blue, EC_50_ = 991 ± 131 μM, *n* = 8), and His521Ala/Arg539Lys rat TRPV2 (green, EC_50_ = 1753 ± 125 μM, *n* = 6). Data are shown as mean ± S.E.M. Current amplitudes were determined for each concentration and normalized to the maximal amplitude obtained with 3000 μM 2-APB. The solid lines represent fits calculated with the Hill equation. Source data are provided as a Source Data file.

The TRPV2_2APB_IAC_ structure revealed a putative 2-APB binding site between S5 of one TRPV2 monomer and the S4-S5 linker of the adjacent (Fig. 2A, B, Supplementary Fig. 4). The proposed TRPV2 2-APB binding site is located at the N-terminal end of the S4-S5 linker, where the ligand coordinates with Arg539, Thr522, His521, and Tyr525 (Fig. 2A, B). In the rat TRPV2_Apo1_^21^, Arg539, His521, and Tyr525 form a network of cation-π interactions, which stabilize the interaction between the S4-S5 linker and S5 from an adjacent monomer (Fig. 2C). 2-APB binding disrupts this network and may act as a wedge to force apart two adjacent TRPV2 monomers at the interface between the S4-S5 linker and the bottom of S5 (Fig. 2A, B, Supplementary Fig. 4). To validate the position and orientation of the 2-APB molecule in the binding site we used RosettaLigand docking^29,30^. The top-scored docking model was in the same location and similar orientation as in the initial fitted model with the reported all-atom root-mean-square deviation (RMSD) of 2.106 Å between the two positions of the drug (Fig. 2D, Supplementary Fig. 5).

To further validate this binding site for 2-APB, we performed whole-cell patch clamp recordings on HEK293T cells transiently transfected with either the wildtype rat TRPV2 or with TRPV2 mutants carrying single-point mutations around the proposed binding site: His521Ala, His521Asn, Thr522Ala, Tyr525Ala, Arg535Lys, and Arg539Lys. As histidine and arginine residues were critical for the TRPV3 consensus 2-APB binding site, we followed the previously established substitutions for these residues^26^. Increasing concentrations of 2-APB, from 30 up to 5000 µM, were applied to the cells in order to obtain dose–response curves for 2-APB-evoked activation for each construct (Fig. 2E–J, Supplementary Fig. 6). The calculated EC_50_ values are summarized in Table 2. When compared to the wildtype TRPV2 (Fig. 2E, Table 2), the 2-APB sensitivity in the His521Ala, His521Asn, and Arg539Lys was reduced (Fig. 2F–H, Table 2) and the double mutant His521Ala/Arg539Lys showed a fivefold decrease in 2-APB sensitivity (Fig. 2I, Table 2). This reduction is comparable to the loss of 2-APB sensitivity previously reported for TRPV3 upon mutation of His426 or Arg696^26^. All mutants involving His521 and Arg539 responded normally to high heat (Supplementary Fig. 6), demonstrating that the mutated channels were functional and that the effect of these mutations was specific to 2-APB. Interestingly, the Thr522Ala and Arg535Lys mutations caused a mild increase in 2-APB sensitivity (Supplementary Fig. 6, Table 2). Finally, Tyr525Ala seemed to have a global loss-of-function phenotype as we could not evoke any membrane currents with either 2-APB or heat. These data strongly support a potential interaction of 2-APB and rat TRPV2 via the proposed binding site and suggest a key role of His521 and Arg539 in the binding.

**Table 2:** Summary of whole-cell patch clamp recordings on HEK293T cells transiently transfected with wildtype or mutant rat TRPV2.

TRPV2 variant	EC_50_, μM	Hill coefficient	Number of experiments
Wildtype	322 ± 27	3.0 ± 0.2	8
His521Ala	991 ± 131	3.3 ± 0.3	8
His521Asn	742 ± 14	4.3 ± 0.3	5
Arg539Lys	683 ± 78	5.6 ± 1.1	4
Arg535Lys	79 ± 11	3.3 ± 0.5	4
Thr522Ala	232 ± 43	3.0 ± 0.3	8
His521Ala/Arg539Lys	1753 ± 125	5.2 ± 0.6	6

The 2-APB binding site determined here for rat TRPV2 is unique to TRPV2 channels. Interestingly, it features the same key amino acids as the consensus 2-APB binding site in mouse TRPV3—histidine and arginine^26^. His521 is highly conserved in mammalian TRPV2, including human TRPV2 (Supplementary Fig. 1B), supporting the recent electrophysiology data on human TRPV2 activation by 2-APB^31^ which differs from earlier calcium flux studies^32,33^. Notably His521 is not conserved in other human TRPV channels (Supplementary Fig. 1A). This mirrors the uniqueness of the 2-APB binding site in TRPV3, which features a conserved His426 for mammalian TRPV3^26^ (Supplementary Fig. 1A). Together these results are in full agreement with previous observations that TRPV1 may yield yet another specific 2-APB binding site^26^.

### Simultaneous binding of 2-APB and CBD to TRPV2

**Fig. 3:**
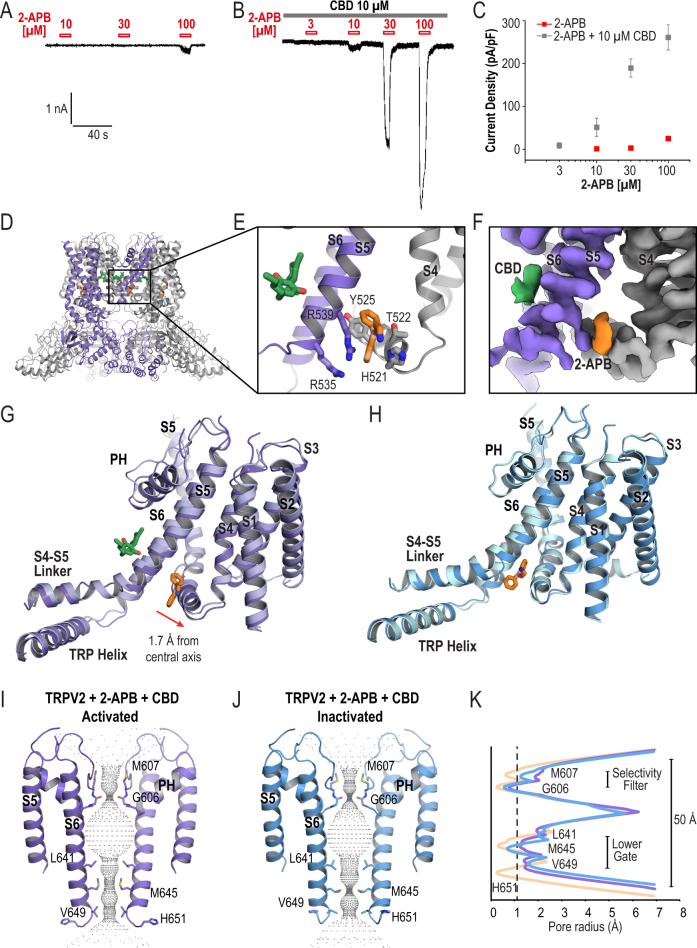
Simultaneous binding of 2-APB and CBD to TRPV2. Representative whole-cell patch clamp current traces displaying an activation of wildtype rat TRPV2 by **A** 10, 30, and 100 μM of 2-APB alone. **B** 10 μM of CBD alone, and synergetic effect of the two agonists applied together. **C** Comparison of current densities; data is shown as mean ± S.E.M. Error bars in the 2-APB only graph are too small to be seen. *N* = 7 for 2-APB alone and *N* = 5 for the two agonists applied together. Source data are provided as a Source Data file. Cartoon representation (**D**, **E**) and map density (**F**) of the 2-APB and CBD binding sites in TRPV2_2APB_CBD_AC_. One monomer is colored medium purple, the adjacent monomer is colored gray; 2-APB is colored orange, CBD is colored green. Map contoured at *σ* = 5. **G** Cartoon representations comparing TRPV2_2APB_AC_ (light blue) and TRPV2_2APB_CBD_AC_ (medium purple), the drugs shown are from TRPV2_2APB_CBD_AC_; models were aligned to S6. **H** Cartoon representations comparing TRPV2_2APB_IAC_ (light cyan) and TRPV2_2APB_CBD_IAC_ (medium blue), the drug shown is from TRPV2_2APB_IAC_; models were aligned to S6. HOLE-generated pore profiles of TRPV2_2APB_CBD_AC_ (**I**) and TRPV2_2APB_CBD_IAC_ (**J**). **K** Graphical representation of the pore profiles of TRPV2_Apo1_ (wheat, PDB 6U84), TRPV2_2APB_ CBD_AC_ (medium purple), TRPV2_2APB_CBD_IAC_ (medium blue). The radius of a dehydrated calcium ion is marked by a dotted gray line at 1.1 Å.

Given the proximity of our herein proposed 2-APB binding site to our previously determined CBD binding site^21^, we decided to investigate whether these two activators could bind simultaneously and affect channel activation. Indeed, when applied together, 2-APB and CBD have a synergetic effect on TRPV2 activation as shown by whole-cell patch recordings on HEK293T cells transiently transfected with the wildtype rat TRPV2 (Fig. 3A–C). Application of 2-APB alone evoked small currents with a density of 0.98 ± 0.1 pA/pF, 2.8 ± 0.3 pA/pF, and 24.9 ± 3.7 pA/pF at 10, 30, and 100 μM respectively (Mean ± SEM, *N* = 7, Fig. 3A). At the same time, in combination with 10 μM CBD 3 μM 2-APB was already enough to elicit a current with a density of 8.9 ± 6.6 pA/pF (Fig. 3B); the current density upon co-application of 10 μM CBD with 10, 30, and 100 μM 2-APB was equal to 51.4 ± 21.3 pA/pF, 189.4 ± 21.1 pA/pF, 260.9 ± 29.5 pA/pF respectively (Mean ± SEM, *N* = 5, Fig. 3B). Thus, a combination of two drugs produced ten times greater current density than the current density induced by either of the agonists alone (Fig. 3B, C).

Therefore, we next collected a cryo-EM dataset of rat TRPV2 in lipid nanodiscs incubated with 1 mM 2-APB and 30 μM CBD for 30 min before preparing the grids. The double-drug dataset had similar heterogeneity to that observed in the 2-APB dataset and was again analyzed using the 3D variability tool^27^ from cryoSPARC^28^ (Supplementary Fig. 7). The dataset yielded two states with C4 symmetry: one identified as activated based on the conformation of the cytoplasmic regulatory switch^23^ with a stable C-terminal helix and N-terminal loop at 3.7 Å resolution (TRPV2_2APB_CBD_AC_) and the other identified as inactivated with a C-terminal loop at 3.8 Å resolution (TRPV2_2APB_CBD_IAC_) (Supplementary Fig. 8, Table 1).

The structures of TRPV2_2APB_CBD_AC_ and TRPV2_2APB_CBD_IAC_ confirmed that 2-APB and CBD can bind to TRPV2 simultaneously: both structures revealed non-protein densities at the proposed 2-APB binding site and at the previously established CBD binding site^21^ (Fig. 3B, C, Supplementary Fig. 4). The drug densities were well-resolved in the TRPV2_2APB_CBD_AC_ structure, where we were able to build both 2-APB and CBD in orientations similar to the poses found in the single drug structures (Fig. 3B, C)^21^. Therefore, the double-drug data independently supported the proposed 2-APB binding site.

Our previously published CBD-bound TRPV2_CBD1_ structure^21^, which has an “activated” conformation of the cytoplasmic regulatory switch, was very similar to TRPV2_Apo1_ (0.591 Å Cα RMSD, PDB 6U8A and 6U84). Here, the activated double-drug structure was more similar to TRPV2_2APB_AC_ (Fig. 3D, 0.640 Å RMSD) than to the TRPV2_CBD1_ structure (Supplementary Fig. 9, 1.464 Å Cα RMSD, PDB 6U8A). At the same time, the two inactivated structures with 2-APB were essentially identical to each other (Fig. 3E; 0.494 Å Cα RMSD) as well as to the previously published CBD-bound TRPV2_CBD2_ structure (Supplementary Fig. 9; 0.751 Å Cα RMSD between TRPV2_2APB_IAC_ and TRPV2_CBD2_; 0.761 Å Cα RMSD between TRPV2_2APB_CBD_IAC_ and TRPV2_CBD2_, PDB 6U88) and only differed locally at the drug binding sites. This led us to the conclusion that transition to the activated conformation in both datasets is driven by 2-APB binding rather than CBD binding, with 2-APB acting as a wedge to initiate conformational changes in the channel.

We next analyzed the pore profiles of TRPV2_2APB_CBD_AC_ and TRPV2_2APB_CBD_IAC_ (Fig. 3F–H). Both double-drug structures remained closed at the selectivity filter; the lower gate of the TRPV2_2APB_CBD_AC_ was partially open with a pore radius of 1.2 Å at Met645 compared to 0.3 Å in the TRPV2_Apo1_ and 0.5 Å in the TRPV2_CBD1_ (Fig. 3H, Supplementary Table 1). The TRPV2_2APB_CBD_IAC_ structure was closed at Met645 with a pore radius of 0.7 Å, similar to TRPV2_CBD2_ at 0.8 Å (Supplementary Table 1). However, these radii are likely insufficient for permeation of calcium ions through the hydrophobic pore of TRPV2. In both double-drug structures, the His651 sidechain was rotated out of the pore as in the 2-APB-bound structures.

### The mechanisms of TRPV2 activation and inactivation

**Fig. 4:**
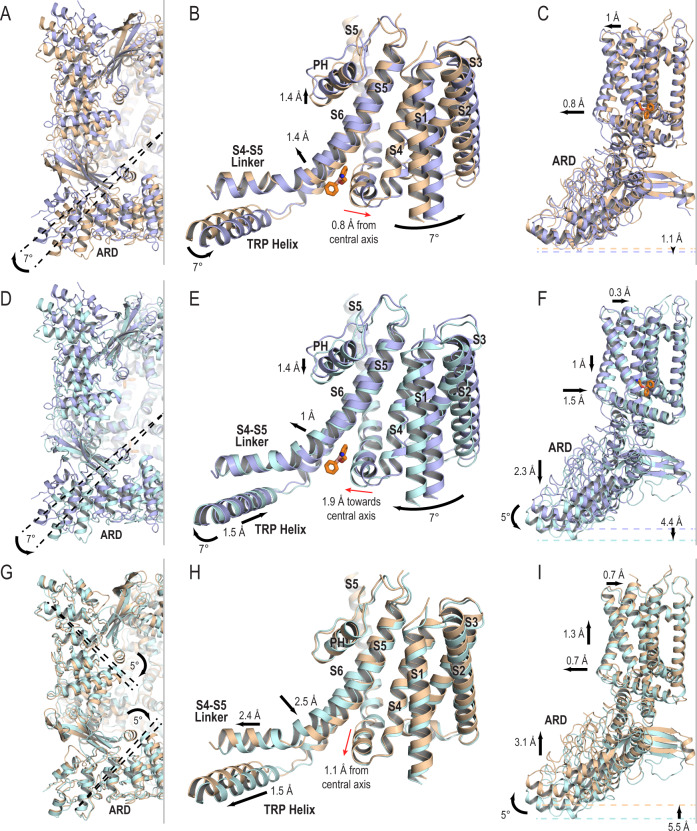
Conformational changes in TRPV2 structure upon 2-APB binding. Cartoon representations comparing TRPV2_Apo1_ (wheat, PDB 6U84), TRPV2_2APB_AC_ (light blue), and TRPV2_2APB_IAC_ (light cyan), the drug shown is from TRPV2_2APB_IAC_; models were aligned to S6. **A**, **D**, **G** View from the cytoplasmic side of TRPV2 showing the rotation of the ARDs as the channel transitions through the gating cycle. **B**, **E**, **H** The channel movements during gating originate from the 2-APB binding site. **C**, **F**, **I** View from the side of TRPV2 showing conformational changes in the TMD and ARDs through the gating cycle.

To understand the gating cycle upon 2-APB binding, we next compared the conformational changes observed between TRPV2_Apo1_ and TRPV2_2APB_AC_ (Fig. 4A–C, Supplementary Movie 2), TRPV2_2APB_AC_ and TRPV2_2APB_IAC_ (Fig. 4D–F, Supplementary Movie 2), and TRPV2_2APB_IAC_ and TRPV2_Apo1_ (Fig. 4G–I, Supplementary Movie 2), aligned with respect to the pore-forming S6 helices (residues 618–648). These assessments revealed that TRPV2 gating could be described as the opening and closing of an iris aperture. To transition from the TRPV2_Apo1_ closed state to the TRPV2_2APB_AC_ open state, the entire assembly except the pore domain (S5-PH-S6) rotated clockwise around the central pore axis by 7° (if viewed from the cytoplasmic side of the protein) (Fig. 4A, B). This large movement of the entire channel originated from a 0.8 Å shift of the S4–S5 linker away from the central pore axis (Fig. 4B). In the pore domain, S5 and the pore helix shifted upwards by 1.4 Å (Fig. 4B), leading to the opening of the selectivity filter together with rotation of Met607 out of the pore (Fig. 1A, B). The expansion of the lower gate was achieved through the widening of the entire transmembrane domain by 0.8–1.0 Å (Fig. 4C), which led to a rotation of His651 at the bottom of S6 out of the pore (Fig. 1A, B). Finally, the ankyrin repeat domains (ARDs) show a slight downward movement of the channel by 1.1 Å (Fig. 4C).

On the contrary, to transition from the activated to the inactivated state, the entire assembly except the pore domain (S5-PH-S6) rotated counterclockwise around the central pore axis by 7° (if viewed from the cytoplasmic side of the protein) (Fig. 4D, E). The S4-S5 linker and the TRP helix rotated and simultaneously shifted by 1.5 Å towards the central pore axis (Fig. 4E) as part of the narrowing of the entire transmembrane domain of the channel (Fig. 4F) which closed the lower gate of the pore (Figs. 4F, 1C, 1F). The selectivity filter closed through a downward shift of S5 and the pore helix by 1.4 Å (Fig. 4F). Finally, in the inactivated state there was a significant 4.4 Å downward movement of the ARDs (Fig. 4F), caused by the combination of a downward shift of 2.3 Å and a 5° pivot of each individual ARD (Fig. 4F). The ARDs pivoting coincided with the conformational changes in the cytoplasmic regulatory switch (Fig. 1H, I). In order to come back to the TRPV2_Apo1_ closed state from the 2-APB-bound TRPV2_2APB_IAC_ inactivated state, the drug needs to leave the binding pocket allowing the structure to relax the entire transmembrane domain and ARDs to their original positions (Fig. 4G–I).

The double-drug structures displayed similar movements throughout the gating cycle (Fig. 3G, H, Supplementary Fig. 9). Comparing to TRPV2_Apo1_, the TRPV2_2APB_CBD_AC_ state displayed the same 7° rotation around the central pore axis as TRPV2_2APB_AC_ and a 1.7 Å shift of the S4-S5 linker away from the central pore (Fig. 3G). While the shift of the S4-S5 linker was larger in TRPV2_2APB_CBD_AC_ than in TRPV2_2APB_AC_, this is likely the result of local accommodation of CBD as the overall expansion of the transmembrane domain did not increase. The other significant difference between the two structures was observed at the top of the pore, where in the double-drug structure the pore helix did not move up as much as in the 2-APB-bound one (Fig. 3G) and therefore the selectivity filter of the double-drug structure remained closed (Fig. 3D, F). This is primarily because of the presence of CBD, which stabilizes S5 and does not allow for its upward movement and thus for propagation of conformational changes upon 2-APB binding all the way up to the pore helix. The inactivated structures TRPV2_2APB_IAC_ and TRPV2_2APB_CBD_IAC_ were mostly identical and only differed at the drug-binding sites with small local adjustments (Fig. 3H). Therefore, we concluded that 2-APB-bound and double-drug structures represent the same functional states of the channel.

**Fig. 5:**
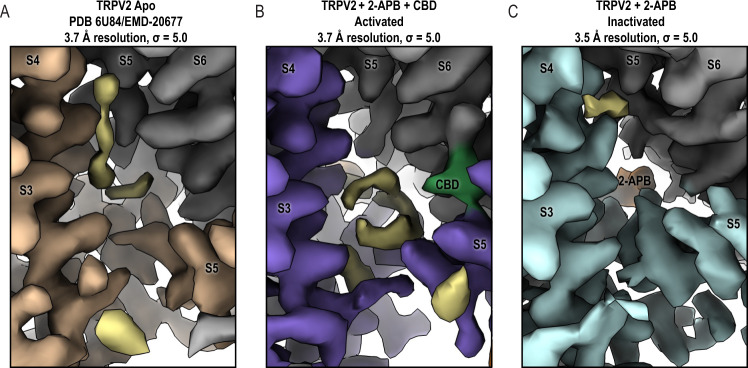
Expulsion of the lipid from the vanilloid pocket of TRPV2 upon 2-APB binding. **A** The vanilloid pocket of TRPV2_Apo1_ (PDB 6U84) contains well-resolved lipid tails. **B** TRPV2_2APB_CBD_AC_ has disrupted lipid density in the vanilloid pocket. **C** TRPV2_2APB_IAC_ displays minimal density that could be attributed to the lipid tails in the vanilloid pocket. One monomer is colored wheat, medium purple or light cyan, the adjacent monomer is colored gray; 2-APB is colored orange, CBD is colored green, potential lipid densities are colored khaki. Maps contoured at *σ* = 5.

The double-drug structures were also complimentary to the 2-APB structures when it comes to analysis of the lipid density in the vanilloid pocket as part of the TRPV2 activation mechanism. In the closed TRPV2_Apo1_ state, as well as in TRPV2_Apo2_^21^ and TRPV2_CBD1_^21^, the vanilloid pocket contained well-resolved lipid tails with no definitive head group (Fig. 5A, Supplementary Fig. 10). We compared these lipid tail densities with the corresponding non-protein densities in the vanilloid pockets of the best 2-APB-bound structures: activated TRPV2_2APB_CBD_AC_ and inactivated TRPV2_2APB_IAC_ (Fig. 5). In TRPV2_2APB_CBD_AC_, the lipid underwent significant conformational re-arrangement with the lipid resting along the S4-S5 linker rather than extending upwards along S4 (Fig. 5B, Supplementary Fig. 10). On the other hand, TRPV2_2APB_IAC_ had minimal lipid density, similar to our previously solved inactivated CBD-bound structure TRPV2_CBD2_^21^, and the cryo-EM map was of sufficient quality to resolve the lipid if it were present (Fig. 5C, Supplementary Fig. 10). The other two 2-APB-bound states, TRPV2_2APB_AC_ and TRPV2_2APB_CBD_IAC_, were consistent with the structures described above (Supplementary Fig. 10). Therefore, we concluded that the vanilloid lipid may play a key role in 2-APB-based TRPV2 channel gating, similar to the previously proposed mechanism for TRPV1 activation by small molecules^34^.

## Discussion

In the present study we were able to determine structures of 2-APB-bound TRPV2 embedded in lipid nanodiscs in two distinct states, which provided a unique opportunity to analyze modulation of this channel by 2-APB in a near-native environment. TRPV1–TRPV3 are structurally homologous, share at least 38% sequence identity and can be activated by 2-APB^22^, thus it is logical to assume that they share a common 2-APB interaction site. However, there is compelling evidence that 2-APB binding sites could be unique to each channel. In 2009, the Patapoutian lab used a high-throughput mutagenesis screen of mouse TRPV3 to identify His426 and Arg696 as crucial for effective activation by 2-APB^26^. Notably, His426 is not conserved in TRPV1 or TRPV2 and introducing it at that location did not change the effect of 2-APB on either channel^26^. In 2010, the Vlachova lab used targeted mutation of the rat TRPV1 S4-S5 linker to identify mutations that reduced 2-APB sensitivity without loss of heat or capsaicin sensitivity, including Lys571Glu^35^. They made the analogous mutations in TRPV2 and TRPV3 and did not observe any loss of 2-APB sensitivity^35^. In 2018, the Sobolevsky lab used cryo-EM to further explore mouse TRPV3 activation by 2-APB^25^. They identified three interaction sites for 2-APB, including the previously characterized His426/Arg696 site^25^. The other two binding sites were not observed in the subsequent cryo-EM studies of 2-APB-bound TRPV3^23,24^, nor in the 2-APB-bound TRPV2 structures presented in this paper (Supplementary Fig. 11). However, one of these additional 2-APB binding sites was identified as the 2-APB binding site in TRPV6 where 2-APB acts as an inhibitor^36^. Overall, these previous studies show that the interaction of 2-APB with TRPV1-TRPV3 is not based around a single shared site^23–26,35^.

Our cryo-EM data allows us to propose a single novel site for 2-APB binding to TRPV2 between S5 and the S4-S5 linker of an adjacent monomer (Fig. 2). In silico docking supports the putative 2-APB binding site and the orientation of the drug. Using electrophysiology, we verified the key interacting residues by mutations but were not able to completely abolish TRPV2 sensitivity to 2-APB. Notably, the previous studies to identify the 2-APB binding site in TRPV3 were also not able to completely abolish the response to 2-APB^25,26^. This may indicate that TRPV2 contains more than one 2-APB binding site, as was proposed for TRPV3 by the Sobolevsky lab, but we observe only one potential location for 2-APB in our current cryo-EM maps.

To date, our TRPV2 structures in the presence of CBD^21^ or 2-APB in a lipid environment have revealed two unique locations for activator binding at the dynamic S4-S5 linker region, sometimes referred to as the “gearbox” of TRP channels^37^. This data argues for the S4-S5 linker as a rich target area for development of both TRPV2-specific activators and inhibitors. Despite the close proximity of the binding sites, the two drugs can bind to TRPV2 simultaneously (Fig. 3) and function synergistically. The addition of CBD appears to have a stabilizing effect on the activated state as seen from the larger proportion of particles and improved resolution of the activated structure in the double-drug dataset. The proposed mechanism of 2-APB action as a wedge further narrows down the region of interest for the rational design of TRPV2-specific drugs to the N-terminal part of the S4-S5 linker.

Abundant cryo-EM and X-ray crystallography data from TRPV1-TRPV3 has become available in the last decade^15–21,23–25,34,38–48^, but comparison of those channels and identification of structural features functionally relevant for native channel gating remain difficult as different orthologous proteins with sequence modifications were used and the majority were purified in detergents/amphipols for structural studies. Recently, the structures of full-length TRPV1-TRPV3 have been solved in nanodiscs^19,21,24,43,46–48^ closely mimicking the native membrane environment and providing the opportunity for their most accurate comparison. Based on this wealth of information, there are a few key common features observed in the gating cycle that are conserved between TRPV1-TRPV3. First, the cytoplasmic regulatory switch at the C-terminus of the channel must be in a helical conformation for channel activation, while a loop conformation of the C-terminus prevents channel opening^23,46,47^ (Supplementary Fig. 12). Second, channel activation includes a 4–8° rotation of the ARD and TRP helix around the central pore^46–48^ (Supplementary Fig. 12), while channel inactivation coincides with a downwards movement of the ARDs^46,47^. Third, the selectivity filter of TRPV1-TRPV3 is not an activation gate and can flexibly accommodate a range of ions^48,49^.

In the current study, we obtained structures of native rat TRPV2 in lipid membranes in the presence of the activator 2-APB alone or together with CBD, resolving a gating cycle of TRPV2. The global movements upon 2-APB activation of TRPV2 are the same as observed in drug and heat activation of TRPV1 and TRPV3: 2-APB binding wedges apart two monomers of TRPV2, rotating the channel 7° around the central pore, disrupting the vanilloid lipid, and initiating pore opening. In the inactivated states, the channel rotated back by 7° and the cytoplasmic regulatory switch transitioned from a helix to a loop conformation, allowing the ARDs to move downwards and closing the channel, but the drug remained bound and the vanilloid lipid was absent.

While the global channel movements of the activated TRPV2 states align with those of the most open TRPV1 and TRPV3 structures (Supplementary Fig. 12), we also need to look at the pore to determine whether or not the channel is open. The selectivity filter in the two activated states varies (Figs. 1, 3), presumably due to the inherent flexibility of this region of TRPV2. The pore radius of the channel at the lower gate of the TRPV2_2APB_AC_ is 1.9 Å, which would allow passage of a dehydrated ion (1.1 Å for calcium), but not for a fully hydrated ion (4.1 Å for calcium). The hydration state of ions passing through TRPV1-TRPV3 is unclear, but several structures of TRPV1 and TRPV3 have a lower gate with a minimum radius greater than 1.1 Å (Supplementary Fig. 13, Supplementary Table 1) and their interpretation falls along a spectrum from “sensitized” or “intermediate” to fully open. Based on a comparison to the other published TRPV1^47,48^ and TRPV3^46^ structures, we interpret the 2-APB-activated TRPV2 structures as intermediates on the path to opening. Nevertheless, the pore radius in even the most open of the TRPV1-TRPV3 structures is still too narrow to allow fully hydrated ions to pass^50^.

With the extensive progress in cryo-EM imaging and data processing achieved over the last few years, more work can now be done to characterize native modulators of channel activity and for drug discovery.

## Methods

### Protein sequence alignment of the TRPV channels

TRPV sequence alignments were performed using Clustal Omega^51^. Graphics of sequence alignments were generated using Aline^52^.

### Protein expression and purification

Full-length rat TRPV2 was expressed and isolated as previously published^21^. Briefly, rat TRPV2 cloned into a YepM vector and tagged with 1D4 was expressed in Saccharomyces cerevisiae. The yeast expressing TRPV2 were lysed in 25 mM Tris, pH 8.0, 300 mM Sucrose, and 5 mM EDTA using a M110Y Microfluidizer (Microfluidics), and the membranes isolated by ultracentrifugation at 100,000 x *g*. These TRPV2-enriched membranes were solubilized in 20 mM Hepes, pH 8.0, 150 mM NaCl, 5% glycerol, 0.087% LMNG, 2 mM TCEP, and 1 mM PMSF for 1 h. This mixture was clarified by centrifugation at 100,000 x *g* and the insoluble fraction discarded. The solubilized TRPV2 was bound to 1D4 antibody coupled CnBr-activated Sepharose beads, followed by washes with Wash Buffer (20 mM Hepes, pH 8.0, 150 mM NaCl, 2 mM TCEP) supplemented with 0.006% (w/v) DMNG. The protein was eluted with Wash Buffer supplemented with 0.006% (w/v) DMNG and 3 mg/mL 1D4 peptide. The purified TRPV2 was concentrated to a volume under 1 mL and reconstituted into nanodiscs in a 1:1:200 ratio of TRPV2:MSP2N2:soy polar lipids (Avanti). The soy polar lipids were rapidly dried under a nitrogen flow and further dried under vacuum before being resuspended in Wash Buffer containing DMNG in a 1:2.5 ratio (soy polar lipids:DMNG). The assembled nanodisc reconstitution mixture was incubated at 4 °C for 30 min before adding Bio-Beads to the mixture. After 1 h, the reconstitution mixture was transferred to a new tube with fresh Bio-Beads and incubated overnight at 4 °C. The nanodisc embedded TRPV2 was purified from empty nanodiscs using a Superose 6 column (GE) equilibrated in Wash Buffer for size-exclusion chromatography. The eluted TRPV2 was concentrated to 2 mg/mL to use in vitrification.

MSP2N2 was expressed and purified as previously described^53^. Briefly, MSP2N2 inserted into a pET28a vector (Addgene) was expressed in BL21 (DE3) cells. After harvest, the cells were resuspended in a buffer containing 20 mM Tris-HCL, pH 7.4, 1 mM PMSF, and a complete EDTA-free protease inhibitor cocktail tablet (Roche) and lysed using a M110Y Microfluidizer (Microfluidics). The lysate supernatant was bound to Ni-NTA resin, which was then washed with Wash Buffer (20 mM Tris-HCl, pH 7.4, 100 mM NaCl), then by Wash Buffer supplemented with 1% Triton X-100, then by Wash Buffer supplemented with 50 mM sodium cholate, then by Wash Buffer supplemented with 20 mM imidazole. MSP2N2 was then eluted from the Ni-NTA resin with Wash Buffer supplemented with 300 mM imidazole and exchanged into 50 mM Tris-HCl, pH 7.5, 100 mM NaCl, 5 mM EDTA before being concentrated to ~10 mg/mL.

### Cryo-EM sample preparation

For 2-APB-bound TRPV2, 1 mM 2-APB was incubated with TRPV2 for 5 min on ice prior to grid preparation. For the 2-APB and CBD-bound sample, 1 mM 1-APB and 30 μM CBD were incubated with TRPV2 for 30 min prior to grid preparation. Immediately before blotting, 3 mM fluorinated Fos-choline 8 (Anatrace) was added to improve particle distribution in vitreous ice. 3 μL of sample was applied to a freshly glow discharged 200 mesh Quantifoil 1.2/1.3 grid and then blotted for 5–8 s at 4 °C and 100% humidity before vitrification in liquid ethane in Vitrobot Mark IV (FEI).

### Cryo-EM data processing

For the 2-APB dataset, the movies were motion corrected the using the Relion 3.1^54^ implementation of MotionCor2, binning the pixels to 1.06 Å/pix. The remaining processing was done using cryoSPARC v3.2. CTF fit of the motion corrected micrographs was estimated using patch CTF. The dataset was split into two sets due to the large size—15,237 micrographs in set 1, 19,579 micrographs in set 2. The micrographs in each set were curated to remove suboptimal micrographs, resulting in 13,597 micrographs in set 1 and 18,768 micrographs in set 2. From these sets, template-based auto-picking yielded an initial set of 1,928,671 and 2,517,442 particles, respectively. These particles were binned by 4 during extraction and subjected to multiple rounds of 2D classification to remove false positives and bad particles, reducing the particle count to 732,794 particles in set 1 and 1,125,082 particles in set 2. Both sets of particles were reextracted binned by 2 and subjected to multiple rounds of heterogeneous refinement with the TRPV2_Apo1_ map (EMD-20677) as a good reference class and an unrelated map as a reference for noise classes. For set 1/2, this removed 192,713/239,710 particles that did not align to TRPV2 classes and 138,471/258,949 particles that aligned to a TRPV2 class with a completely disrupted pore domain, leaving 401,610/626,423 particles in the classes with the best features. These particles were reextracted to their final box size and refined by Non-uniform refinement with C4 symmetry before symmetry expansion. These particles were then subjected to 3D variability analysis^27^, using a mask that excluded nanodisc density and searching for three modes of movement. The resulting modes were primarily assessed based on the cytoplasmic regulatory switch, as it is a well resolved region with a binary variable. Mode 1 from set 1 and Mode 2 from set 2 represented a C4 transition between a state with the C-terminal helix and a state with the C-terminal loop, while the other modes showed a C2 transition between rotated states with two monomers with the C-terminal helix and 2 with the C-terminal loop. To extract C4 particles with the C-terminal helix: Set 1 particles with Mode 1 < −25, but Mode 0 and 2 between −50 and 50, Set 2 particles with Mode 2 < −25, but Mode 0 and 2 between −50 and 50. To extract C4 particles with the CTD loop: Set 1 particles with Mode 1 > 25, but Mode 0 and 2 between −50 and 50, Set 2 particles with Mode 2 > 25, but Mode 0 and 1 between −50 and 50. After combining the equivalent states from the two sets, there were 486,451 symmetry expanded particles in the state with the C-terminal helix and 486,812 symmetry expanded particles in the state with the C-terminal loop. 3D variability was run on each set again using the same parameters to further clean up the particles. For the particles with the C-terminal loop, the extremes of different modes all showed variation away from the average C4 state, so only the extreme particles (>50 or <−50) in each state were excluded. For the particles with the C-terminal helix, Mode 0 showed a transition between C4 particles with an intact pore domain and particles with a disrupted pore domain, while the other modes described a break from C4 symmetry. Only particles at the most intact end of Mode 0 were taken (>50), and the extreme particles from the other modes were excluded (> 50 or <−50). After reducing symmetry expansion, this resulted in 92,948 particles in the class with the C-terminal loop and 8,258 particles in the class with the C-terminal helix. These particles were then subjected to global and local CTF refinement and Non-uniform refinement with C4 symmetry. Finally, the particles were locally refined with C4 symmetry and map excluding the nanodisc density. The final pixel size was adjusted to 1.07 Å/pix based on comparison with a TRPV2 ARD crystal structure (PDB 2ETA)^55^. There were not enough particles in a stable state along the other modes of movement to be able to extract a good quality structure, and it is unclear whether these C2 and asymmetric transitions are functionally relevant.

For the 2-APB + CBD dataset, all data processing was done in cryoSPARC v 3.2. The 10,291 movies of the dataset were motion corrected using patch motion correction, binning the particles to 1.06 Å/pix. CTF fit of the motion corrected micrographs was estimated using patch CTF. This set of micrographs was then curated to remove suboptimal data, leaving 9354 micrographs. From this set, template-based auto-picking yielded an initial set of 1,709,112 particles. These particles were binned by 4 during extraction and subjected to two rounds of 2D classification to remove false positives and bad particles, reducing the particle count to 820,578. The particles were reextracted binned by 2 and subjected to two rounds of Ab-Initio Reconstruction, each time searching for four classes. The first round yielded 1 class with good TRP channel features, composed of 379,054 particles. The second round yielded two classes with good TRP channel features, composed of a combined 256,203 particles. These particles were reextracted to their final box size and refined by Non-uniform refinement with C4 symmetry and CTF refinement before symmetry expansion. These particles were then subjected to 3D variability analysis^27^, using a mask that excluded nanodisc density and searching for three modes of movement. The resulting modes were primarily assessed based on the cytoplasmic regulatory switch, as it is a well resolved region with a binary variable. Mode 0 represented a C4 transition between a state with the C-terminal helix and a state with the C-terminal loop, Mode 1 showed a C2 transition between rotated states with two opposite monomers with the C-terminal helix and two with the C-terminal loop, while Mode 2 showed an asymmetric transition between two adjacent monomers with the C-terminal helix and two with the C-terminal loop. To extract the two C4 classes, the 3D variability display in cluster mode searching for seven classes was used to analyze Modes 0 and 1. Two of these clusters corresponded to the most extreme ends of Mode 0, while excluding the most extreme ends of Mode 1. After reducing the symmetry expansion, this yielded 30,563 particles in the state with the C-terminal helix (the activated state) and 33,404 particles in the state with the C-terminal loop (the inactivated state). These particles were then subjected to global and local CTF refinement and Non-uniform refinement with C4 symmetry. The final pixel size was adjusted to 1.07 Å/pix based on comparison with a TRPV2 ARD crystal structure (PDB 2ETA)^55^.

### Model building

For TRPV2_2APB_AC_, the model of TRPV2_APO_2_ (PDB 6U86) was used as a starting model and docked into the map in Coot^56^. For TRPV2_2APB_IAC_, the model of TRPV2_CBD_2_ (PDB 6U88) was used as a starting model and docked into the map in Coot. For TRPV2_2APB_CBD_AC_, the model of TRPV2_CBD1_ (PDB 6U84) was used as a starting model and docked into the map in Coot^56^. For TRPV2_2APB_CBD_IAC_, the model of TRPV2_2APB_IAC_ was used as a starting model and docked into the map in Coot^56^. These models were then iteratively manually adjusted to the map and refined using phenix.real_space_refine from the PHENIX software package^57^.

The ligand restraint files for 2-APB and CBD were generated using the eLBOW tool from the PHENIX software package^58^. Due to the variable quality of drug density in the maps, 2-APB and CBD were only built into TRPV2_2APB_IAC_ and TRPV2_2APB_CBD_AC_.

Pore profiles were generated using Hole^59^. Images of the models and maps for figures were generated using Pymol (Schrödinger), Chimera^60^, and ChimeraX^61^.

### Whole-cell voltage clamp

Nanofectin (PAA, Pasching, Austria) was used to transfect HEK293T cells with WT rat TRPV2, Arg535Lys rat TRPV2, Arg539Lys rat TRPV2, His521Ala rat TRPV2, His521Asn rat TRPV2, His521Ala/Arg539Lys rat TRPV2, Thr522Ala rat TRPV2 and Tyr525Ala rat TRPV2. Mutants were generated according to the instructions of the manufacturer by site directed mutagenesis with the quick-change lightning site-directed mutagenesis kit (Aglient, Waldbronn, Germany). All mutants were sequenced to confirm intended amino acid exchange and to exclude further channel mutations. Cells were cultured at 37 °C with 5% CO_2_ in Dulbecco’s modified Eagle medium nutrient mixture F12 (DMEM/F12 Gibco/Invitrogen, Darmstadt, Germany) and supplemented with 10% fetal bovine serum, (Biochrom, Berlin, Germany).

Whole-cell voltage clamp was performed with an EPC10 USB HEKA amplifier (HEKA Electronik, Lamprecht, Germany), much as previously described^31^. Signals were low passed at 1 kHz and sampled at 2–10 kHz. Patch pipettes were pulled from borosilicate glass tubes (TW150F-3; World Precision Instruments, Berlin, Germany) to give a resistance of about 3 MΩ. Cells were held at −60 mV and all recordings were performed at room temperature. The external solution contained: 140 mM NaCl, 5 mM KCl, 2 mM MgCl_2_, 5 mM EGTA, 10 mM glucose, and 10 mM HEPES, pH 7.4 (adjusted with NaOH). Calcium was omitted in order to avoid desensitization. The standard pipette solution contained: 140 mM KCl, 2 mM MgCl_2_, 5 mM EGTA and 10 mM HEPES, pH 7.4 (adjusted with KOH). A gravity-driven glass multi-barrel perfusion system was used to bath apply solutions. Data acquisition and off-line analyses required Patchmaster/Fitmaster software (HEKA Electronik, Lamprecht, Germany) and Origin 8.5.1 (Origin Lab, Northampton, MA, USA).

### Rosetta docking of 2-APB

The starting structure of TRPV2_2APB_IAC_ was refined using Rosetta relax with electron density constraints (see Rosetta scripts and command lines used in Supplementary Note 1). The initial fitted 2-APB molecule was removed for the refinement. We generated 1000 decoys and selected the top-scoring model for the docking step. OpenEye OMEGA (OpenEye Scientific Software)^62^ was used to generate conformers for 2-APB molecule. The primary amine group on 2-APB was protonated to be consistent with its form at the physiological pH. We used RosettaLigand for docking of 2-APB to the top refined model of TRPV2 (see Rosetta scripts and command lines used in Supplementary Note 2). The details of the RosettaLigand docking algorithm have been previously described^29,30^. Here, we used a sampling radius of 3 Å to perturb 2-APB around the binding pocket identified by the electron density. An initial random perturbation of 0.5 Å was added before the Monte Carlo docking cycles to further randomize the docking process. A total of 5000 docking models were generated. Rosetta ligand binding energy represented by the interface_delta_X term was used to select the top docking model. All-atom RMSD was used to compare all docking models to the initial fitted model (Supplementary Fig. 5).

### Reporting summary

Further information on research design is available in the Nature Research Reporting Summary linked to this article.

## Supplementary information

Supplementary Information

Description of Additional Supplementary Files

Supplementary Movie 1

Supplementary Movie 2

Reporting Summary

## Data Availability

The atomic coordinates and cryo-EM density maps generated in this study have been deposited in the Protein Data Bank and Electron Microscopy Data Bank under the accession codes TRPV2_2APB_AC_ (PDB 7N0N and EMD-24110), TRPV2_2APB_IAC_ (PDB 7N0M and EMD-24109), TRPV2_2APB_CBD_AC_ (PDB 7T37 and EMD-25650), TRPV2_2APB_CBD_IAC_ (PDB 7T38 and EMD-25651). The atomic coordinates and cryo-EM density maps of previously solved TRPV2 structures used in this study are available in the Protein Data Bank and Electron Microscopy Data Bank under the accession codes TRPV2_Apo1_ (PDB 6U84 and EMD-20677), TRPV2_CBD1_ (PDB 6U8A and EMD-20686), TRPV2_CBD2_ (PDB 6U88 and EMD-20682), TRPV2 ARD (PDB 2ETA). Source data are provided with this paper.

## Source data

Source Data

## References

[1] Pumroy RA, Fluck EC, Ahmed T, Moiseenkova-Bell VY (2020). Structural insights into the gating mechanisms of TRPV channels. Cell Calcium.

[2] Peralvarez-Marin A, Donate-Macian P, Gaudet R (2013). What do we know about the transient receptor potential vanilloid 2 (TRPV2) ion channel?. FEBS J..

[3] Uhlén M (2015). Tissue-based map of the human proteome. Science.

[4] Entin-Meer M, Keren G (2020). Potential roles in cardiac physiology and pathology of the cation channel TRPV2 expressed in cardiac cells and cardiac macrophages: a mini-review. Am. J. Physiol. Heart Circ. Physiol..

[5] Katanosaka Y (2014). TRPV2 is critical for the maintenance of cardiac structure and function in mice. Nat. Commun..

[6] Iwata Y, Matsumura T (2019). Blockade of TRPV2 is a novel therapy for cardiomyopathy in muscular dystrophy. Int. J. Mol. Sci..

[7] Link TM (2010). TRPV2 has a pivotal role in macrophage particle binding and phagocytosis. Nat. Immunol..

[8] Leveque M (2018). Phagocytosis depends on TRPV2-mediated calcium influx and requires TRPV2 in lipids rafts: alteration in macrophages from patients with cystic fibrosis. Sci. Rep..

[9] De Clercq K (2021). Mapping the expression of transient receptor potential channels across murine placental development. Cell Mol. Life Sci..

[10] Santoni G (2013). The role of transient receptor potential vanilloid type-2 ion channels in innate and adaptive immune responses. Front. Immunol..

[11] Majhi RK (2015). Functional expression of TRPV channels in T cells and their implications in immune regulation. FEBS J..

[12] Hisanaga E (2009). Regulation of calcium-permeable TRPV2 channel by insulin in pancreatic beta-cells. Diabetes.

[13] Sawatani T, Kaneko YK, Doutsu I, Ogawa A, Ishikawa T (2019). TRPV2 channels mediate insulin secretion induced by cell swelling in mouse pancreatic β-cells. Am. J. Physiol.-Cell Physiol..

[14] Santoni G (2020). The TRPV2 cation channels: from urothelial cancer invasiveness to glioblastoma multiforme interactome signature. Lab. Investig..

[15] Conde J (2021). Allosteric antagonist modulation of TRPV2 by piperlongumine impairs glioblastoma progression. ACS Cent. Sci..

[16] Zubcevic L (2016). Cryo-electron microscopy structure of the TRPV2 ion channel. Nat. Struct. Mol. Biol..

[17] Huynh KW (2016). Structure of the full-length TRPV2 channel by cryo-EM. Nat. Commun..

[18] Zubcevic L, Le S, Yang H, Lee SY (2018). Conformational plasticity in the selectivity filter of the TRPV2 ion channel. Nat. Struct. Mol. Biol..

[19] Zubcevic L, Hsu AL, Borgnia MJ, Lee SY (2019). Symmetry transitions during gating of the TRPV2 ion channel in lipid membranes. Elife.

[20] Dosey TL (2019). Structures of TRPV2 in distinct conformations provide insight into role of the pore turret. Nat. Struct. Mol. Biol..

[21] Pumroy RA (2019). Molecular mechanism of TRPV2 channel modulation by cannabidiol. eLife.

[22] Hu HZ (2004). 2-aminoethoxydiphenyl borate is a common activator of TRPV1, TRPV2, and TRPV3. J. Biol. Chem..

[23] Zubcevic L, Borschel WF, Hsu AL, Borgnia MJ, Lee SY (2019). Regulatory switch at the cytoplasmic interface controls TRPV channel gating. Elife.

[24] Deng Z (2020). Gating of human TRPV3 in a lipid bilayer. Nat. Struct. Mol. Biol..

[25] Singh AK, McGoldrick LL, Sobolevsky AI (2018). Structure and gating mechanism of the transient receptor potential channel TRPV3. Nat. Struct. Mol. Biol..

[26] Hu H, Grandl J, Bandell M, Petrus M, Patapoutian A (2009). Two amino acid residues determine 2-APB sensitivity of the ion channels TRPV3 and TRPV4. Proc. Natl Acad. Sci. USA.

[27] Punjani A, Fleet DJ (2021). 3D variability analysis: Resolving continuous flexibility and discrete heterogeneity from single particle cryo-EM. J. Struct. Biol..

[28] Punjani A, Rubinstein JL, Fleet DJ, Brubaker MA (2017). cryoSPARC: algorithms for rapid unsupervised cryo-EM structure determination. Nat. Methods.

[29] Meiler J, Baker D (2006). ROSETTALIGAND: protein-small molecule docking with full side-chain flexibility. Proteins.

[30] DeLuca S, Khar K, Meiler J (2015). Fully flexible docking of medium sized ligand libraries with RosettaLigand. PLoS One.

[31] Fricke TC (2019). Oxidation of methionine residues activates the high-threshold heat-sensitive ion channel TRPV2. Proc. Natl Acad. Sci. USA.

[32] Neeper MP (2007). Activation properties of heterologously expressed mammalian TRPV2: evidence for species dependence. J. Biol. Chem..

[33] Juvin V, Penna A, Chemin J, Lin YL, Rassendren FA (2007). Pharmacological characterization and molecular determinants of the activation of transient receptor potential V2 channel orthologs by 2-aminoethoxydiphenyl borate. Mol. Pharm..

[34] Gao Y, Cao E, Julius D, Cheng Y (2016). TRPV1 structures in nanodiscs reveal mechanisms of ligand and lipid action. Nature.

[35] Boukalova S, Marsakova L, Teisinger J, Vlachova V (2010). Conserved residues within the putative S4-S5 region serve distinct functions among thermosensitive vanilloid transient receptor potential (TRPV) channels. J. Biol. Chem..

[36] Singh AK, Saotome K, McGoldrick LL, Sobolevsky AI (2018). Structural bases of TRP channel TRPV6 allosteric modulation by 2-APB. Nat. Commun..

[37] Hofmann L (2017). The S4-S5 linker—gearbox of TRP channel gating. Cell Calcium.

[38] Cao E, Liao M, Cheng Y, Julius D (2013). TRPV1 structures in distinct conformations reveal activation mechanisms. Nature.

[39] Liao M, Cao E, Julius D, Cheng Y (2013). Structure of the TRPV1 ion channel determined by electron cryo-microscopy. Nature.

[40] Moiseenkova-Bell VY, Stanciu LA, Serysheva II, Tobe BJ, Wensel TG (2008). Structure of TRPV1 channel revealed by electron cryomicroscopy. Proc. Natl Acad. Sci. USA.

[41] Huynh KW (2014). Structural insight into the assembly of TRPV channels. Structure.

[42] Nadezhdin KD (2021). Extracellular cap domain is an essential component of the TRPV1 gating mechanism. Nat. Commun..

[43] Shimada H (2020). The structure of lipid nanodisc-reconstituted TRPV3 reveals the gating mechanism. Nat. Struct. Mol. Biol..

[44] Zubcevic L (2018). Conformational ensemble of the human TRPV3 ion channel. Nat. Commun..

[45] Singh AK (2019). Structural basis of temperature sensation by the TRP channel TRPV3. Nat. Struct. Mol. Biol..

[46] Nadezhdin KD (2021). Structural mechanism of heat-induced opening of a temperature-sensitive TRP channel. Nat. Struct. Mol. Biol..

[47] Kwon DH (2021). Heat-dependent opening of TRPV1 in the presence of capsaicin. Nat. Struct. Mol. Biol..

[48] Zhang K, Julius D, Cheng Y (2021). Structural snapshots of TRPV1 reveal mechanism of polymodal functionality. Cell.

[49] Jara-Oseguera A, Huffer KE, Swartz KJ (2019). The ion selectivity filter is not an activation gate in TRPV1-3 channels. Elife.

[50] Huffer KE, Aleksandrova AA, Jara-Oseguera A, Forrest LR, Swartz KJ (2020). Global alignment and assessment of TRP channel transmembrane domain structures to explore functional mechanisms. Elife.

[51] Sievers F (2011). Fast, scalable generation of high-quality protein multiple sequence alignments using Clustal Omega. Mol. Syst. Biol..

[52] Bond CS, Schuttelkopf AW (2009). ALINE: a WYSIWYG protein-sequence alignment editor for publication-quality alignments. Acta Crystallogr. D. Biol. Crystallogr..

[53] Hughes TET (2018). Structural insights on TRPV5 gating by endogenous modulators. Nat. Commun..

[54] Zivanov J (2018). New tools for automated high-resolution cryo-EM structure determination in RELION-3. Elife.

[55] Jin X, Touhey J, Gaudet R (2006). Structure of the N-terminal ankyrin repeat domain of the TRPV2 ion channel. J. Biol. Chem..

[56] Emsley P, Lohkamp B, Scott WG, Cowtan K (2010). Features and development of Coot. Acta Crystallogr. D. Biol. Crystallogr..

[57] Afonine PV (2018). Real-space refinement in PHENIX for cryo-EM and crystallography. Acta Crystallogr. D. Struct. Biol..

[58] Moriarty NW, Grosse-Kunstleve RW, Adams PD (2009). electronic Ligand Builder and Optimization Workbench (eLBOW): a tool for ligand coordinate and restraint generation. Acta Crystallogr. D. Biol. Crystallogr..

[59] Smart OS, Neduvelil JG, Wang X, Wallace BA, Sansom MSP (1996). HOLE: A program for the analysis of the pore dimensions of the ion channel structural models. J. Mol. Graph..

[60] Pettersen EF (2004). UCSF Chimera—a visualization system for exploratory research and analysis. J. Comput. Chem..

[61] Goddard TD (2018). UCSF ChimeraX: meeting modern challenges in visualization and analysis. Protein Sci..

[62] Hawkins PCD, Skillman AG, Warren GL, Ellingson BA, Stahl MT (2010). Conformer generation with OMEGA: algorithm and validation using high quality structures from the Protein Databank and Cambridge Structural Database. J. Chem. Inform. Model.

