# Supplementary material for: Structural insights into TRPV2 activation by small molecules
Source: Nat Commun. 2022 Apr 28;13:2334. doi: 10.1038/s41467-022-30083-3 (PMC9051106; doi:10.1038/s41467-022-30083-3)
Supplement: Supplementary file 1 — Supplementary Information [file 41467_2022_30083_MOESM1_ESM.pdf]

Supplementary information for

Structural insights into TRPV2 activation by small molecules

Ruth A. Pumroy*, Anna D. Protopopova*, Tabea C. Fricke, Iris U. Lange, Ferdinand M. Haug, Phuong T. Nguyen, Pamela N. Gallo, Bárbara B. Sousa, Gonçalo J.L. Bernardes, Vladimir Yarov-Yarovoy, Andreas Leffler, Vera Y. Moiseenkova-Bell#

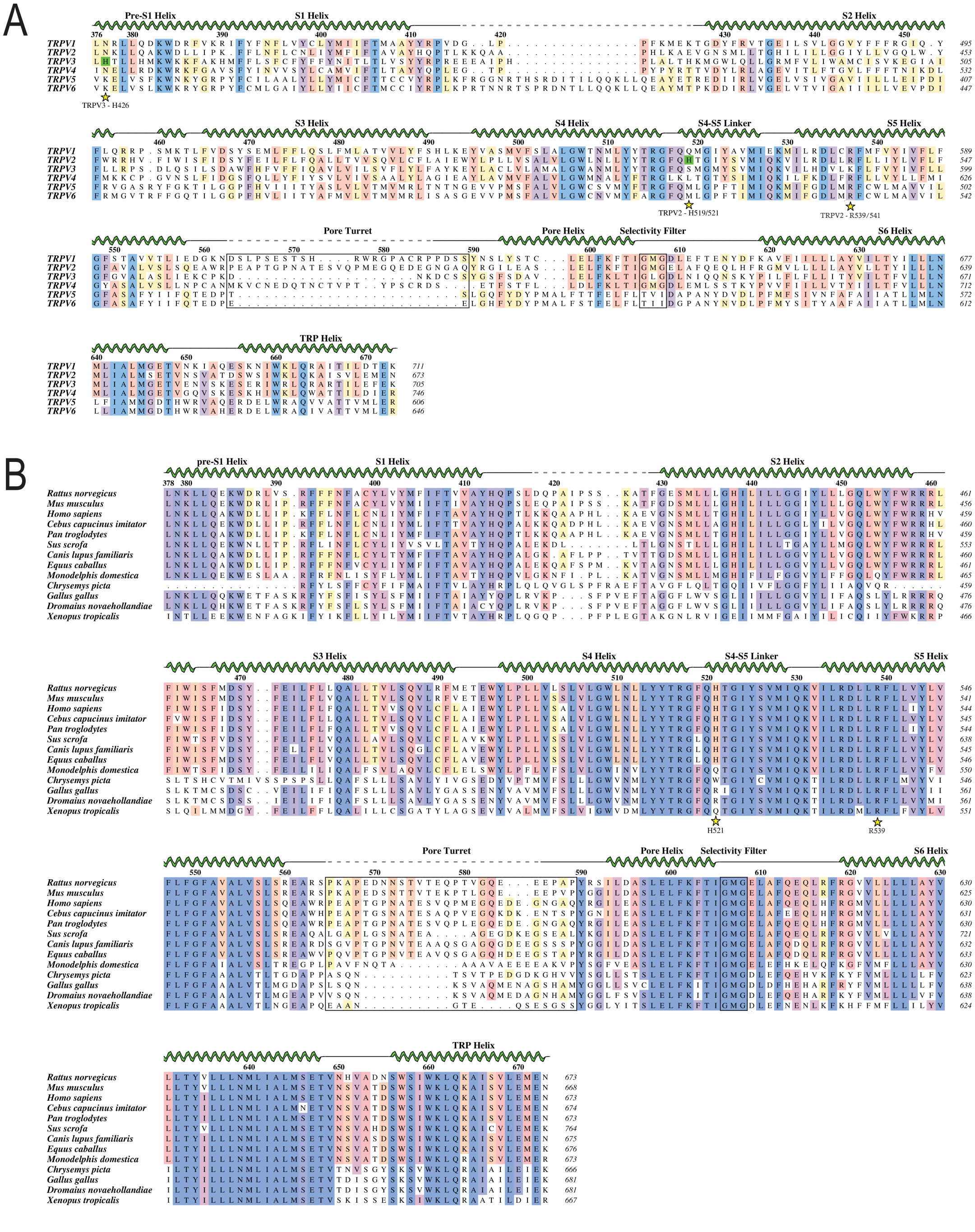

Supplementary Figure 1. Sequence alignments of the TRPV2 transmembrane domain. Sequence alignments of (A) the human TRPV family and (B) a selection of TRPV2 orthologs from the Pre-S1 helix to the TRP helix. The human TRPV family alignment is numbered based on human TRPV2, the TRPV2 ortholog alignment is numbered based on rat TRPV2, residue numbers for each sequence are marked at the end of each line. Residues with conserved identity are highlighted with a gradient from pale yellow (50%) to blue (100%). The key histidine residues involved in 2-APB interaction with TRPV2 and TRPV3 are marked with a green highlight in the human TRPV family alignment. The key TRPV2 residues for 2-APB binding (His521 and Arg539) are labeled and marked with stars.

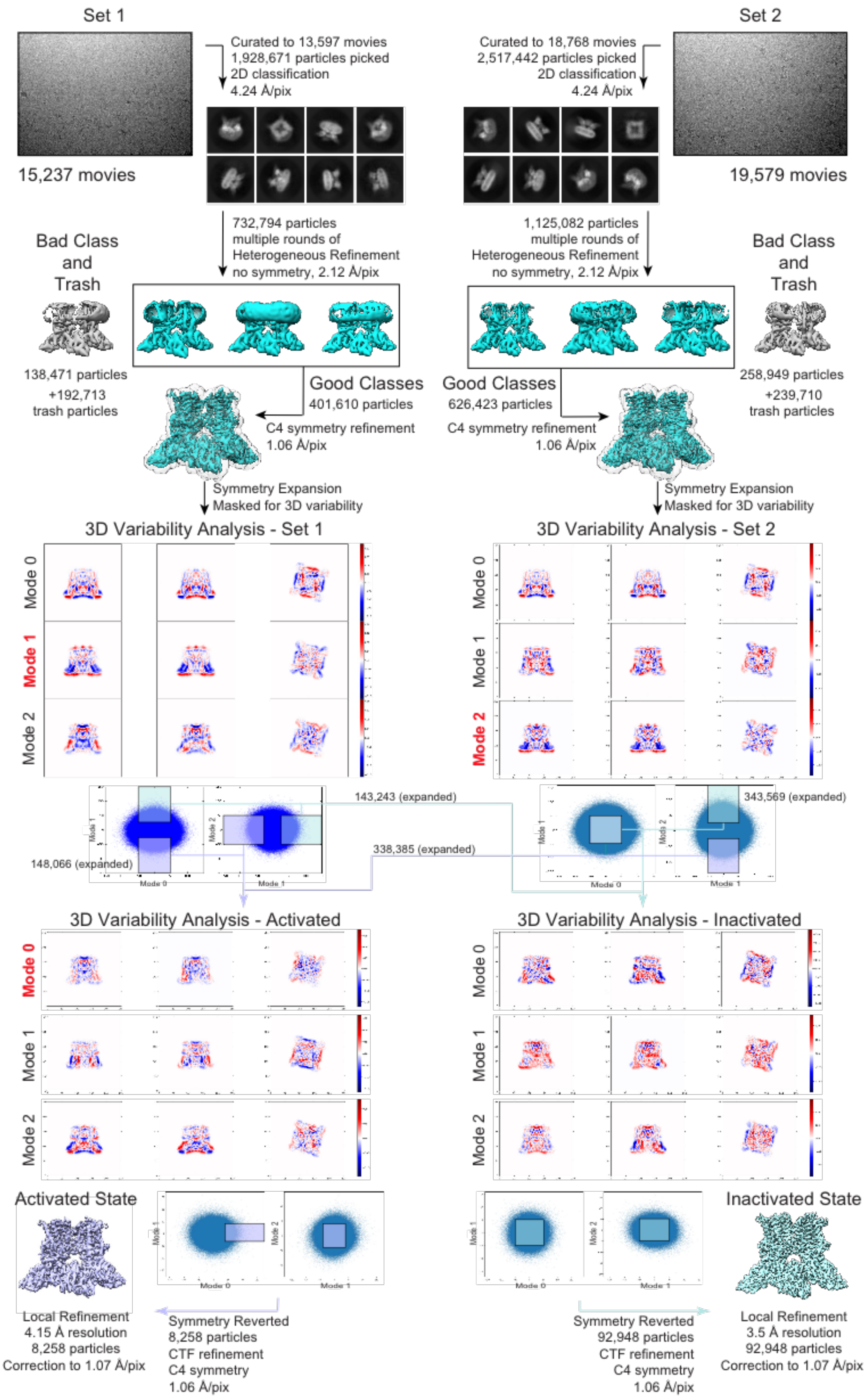

Supplementary Figure 2. Diagram of the TRPV2$_{2APB}$ dataset processing path from cryoSPARC.

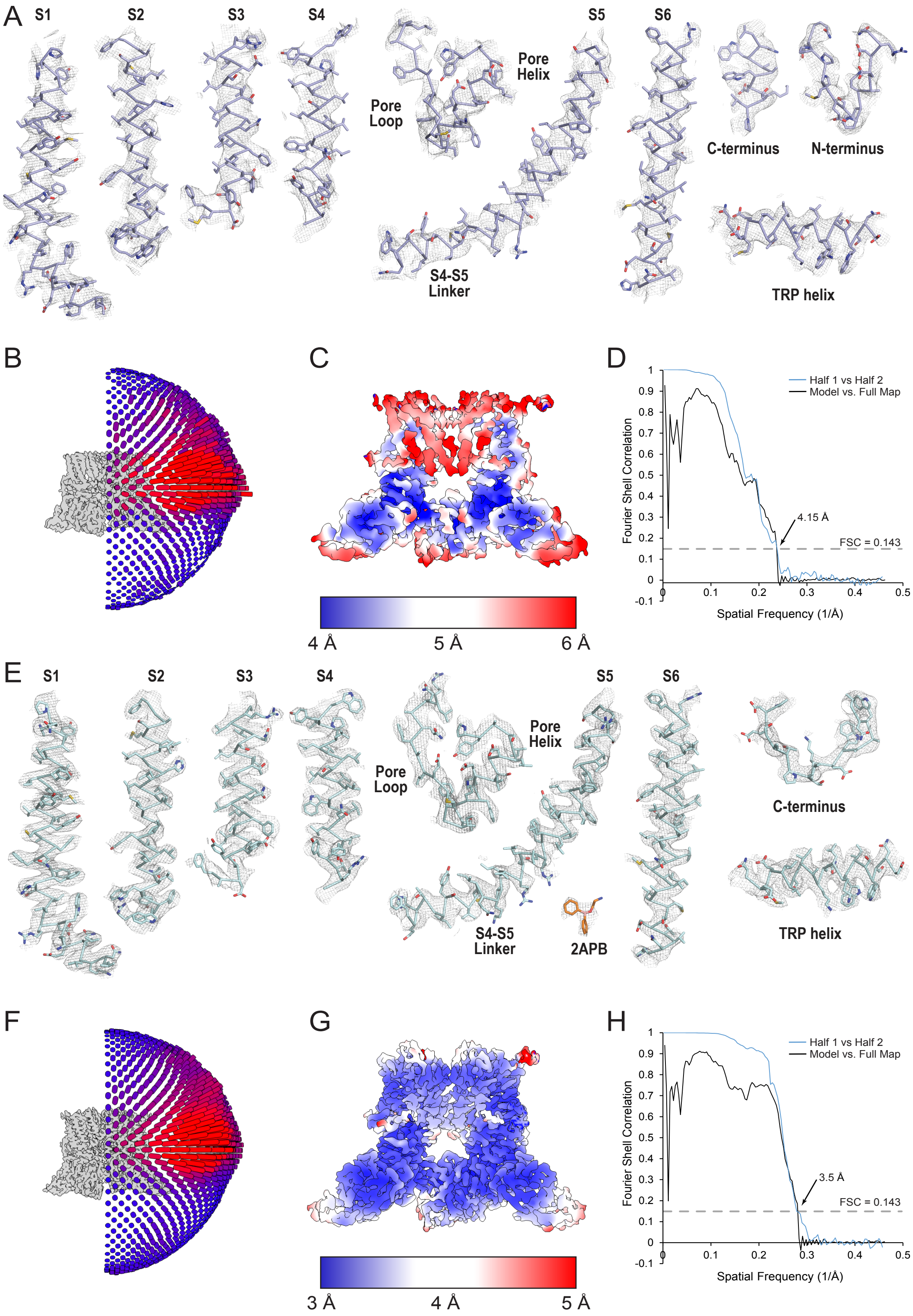

Supplementary Figure 3. Analysis of TRPV2$_{2APB}$ cryo-EM data quality. (A) Representative densities of the TRPV2$_{2APB_AC}$ cryo-EM map. Model is depicted as a ribbon with sidechains as sticks; all densities are contoured at σ = 4.2. (B) Angular distribution of particles in the final TRPV2$_{2APB_AC}$ C4 refinement. (C) Local resolution of the TRPV2$_{2APB_AC}$ map. (D) FSC curves of the final TRPV2$_{2APB_AC}$ map and model validation. (E) Representative densities of the TRPV2$_{2APB_IAC}$ cryo-EM map. Model is depicted as a ribbon with sidechains as sticks; all densities are contoured at σ = 4.5. (F) Angular distribution of particles in the final TRPV2$_{2APB_IAC}$ C4 refinement. (G) Local resolution of the TRPV2$_{2APB_IAC}$ map. (H) FSC curves of the final TRPV2$_{2APB_IAC}$ map and model validation.

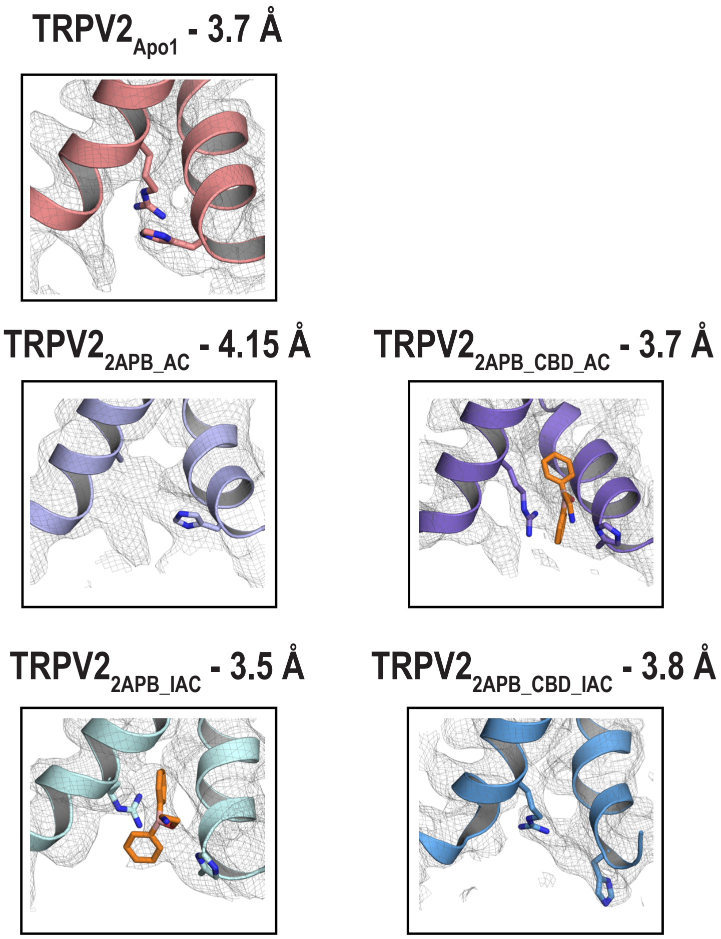

Supplementary Figure 4. Comparison of cryo-EM density maps at the 2-APB binding site of TRPV2$_{Apo1}$, TRPV2$_{2APB_AC}$, TRPV2$_{2APB_IAC}$, TRPV2$_{2APB_CBD_AC}$, and TRPV2$_{2APB_CBD_IAC}$ maps. Models are shown as cartoon with selected sidechains and drug shown as sticks. All density maps are shown as gray mesh contoured at σ = 4.0.

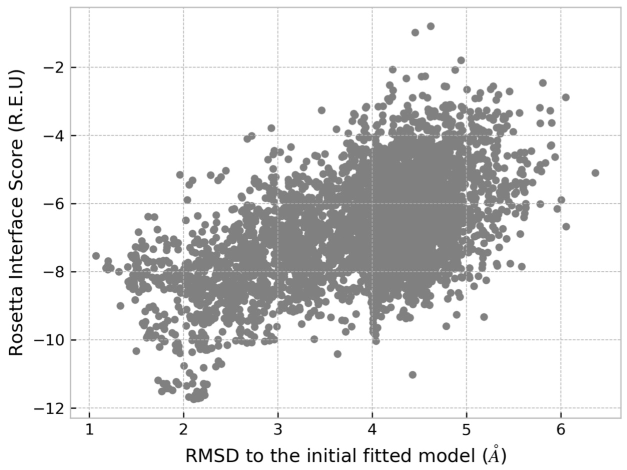

Supplementary Figure 5. Rosetta docking of 2APB. 2-APB docking landscape represented by the Rosetta Interface Score and the all-atom root-mean-square deviation (RMSD) to the initial fitted model. Energy is reported in Rosetta Energy Unit (R.E.U).

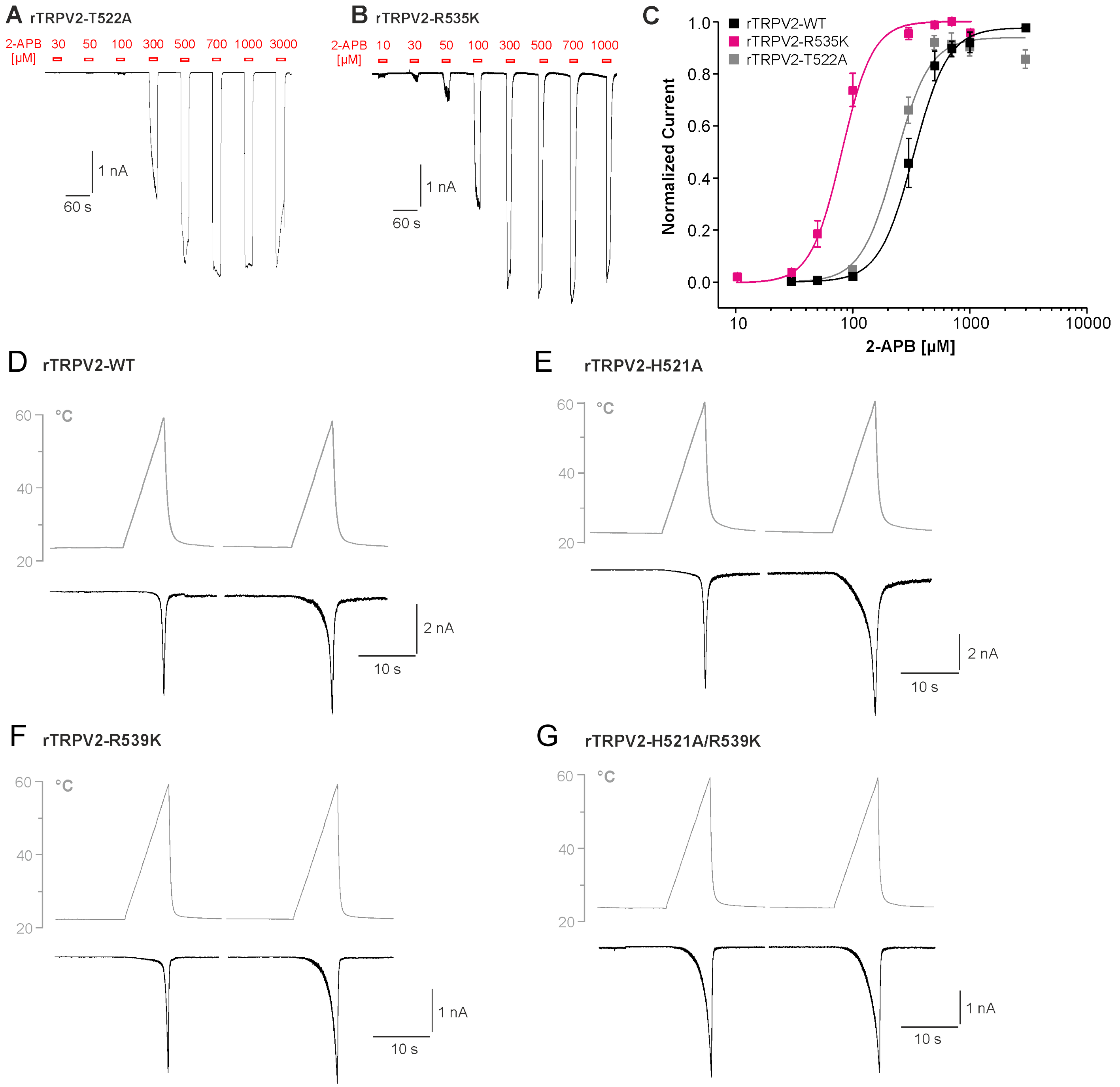

Supplementary Figure 6. Whole-cell patch clamp electrophysiology data on TRPV2 activation with 2-APB and heat. Representative whole-cell patch clamp current traces displaying a concentration-dependent activation of Thr522Ala rat TRPV2 (A), and Arg535Lys rat TRPV2 (B) by 2-APB. Cells were held at -60 mV and increasing concentrations of 2-APB were applied. (C) Dose-response curves for 2-APB-evoked activation of wildtype rat TRPV2 (black, EC_{50}=322±27 μM n=8), Thr522Ala rat TRPV2 (gray, EC_{50}=232±43 μM, n=8), and Arg535Lys rat TRPV2 (pink, EC_{50}=79±11 μM, n=4); data is shown as mean ± S.E.M. Current amplitudes were determined for each concentration and normalized to the maximal amplitude obtained with 1000-3000 μM 2-APB. The solid lines represent fits calculated with the Hill equation. Data Representative whole-cell patch clamp current traces displaying heat-induced activation of wildtype rat TRPV2 (D), His521Ala rat TRPV2 (E), Arg539Lys rat TRPV2 (F), and His521Ala/Arg539Lys rat TRPV2 (G). Cells were held at -60 mV and temperature was ramped from ~24ºC to ~60ºC. Current traces are representative samples from the 3-4 cells recorded from each mutant.

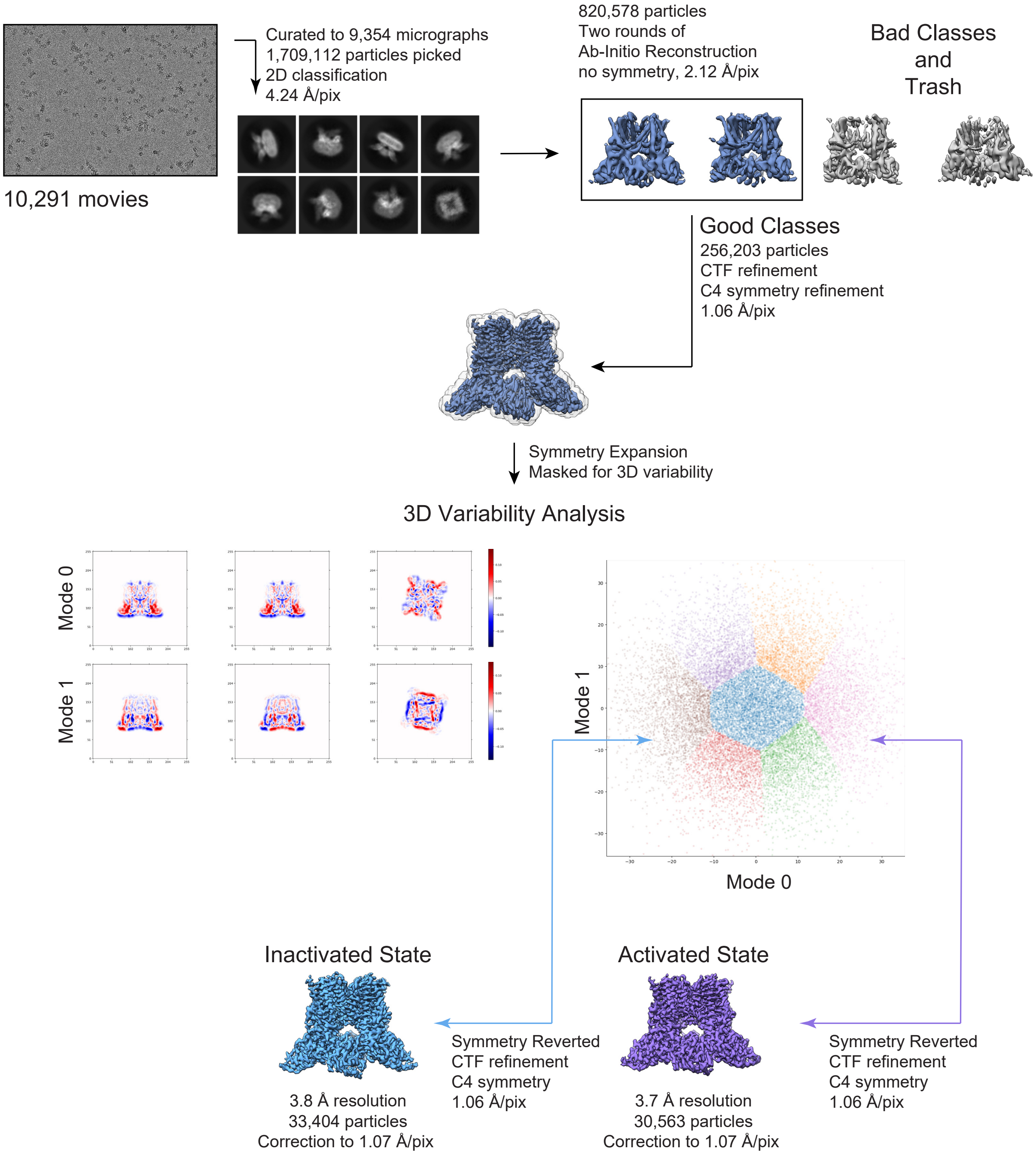

Supplementary Figure 7. Diagram of the TRPV2$_{2APB_CBD}$ dataset processing path from cryoSPARC.

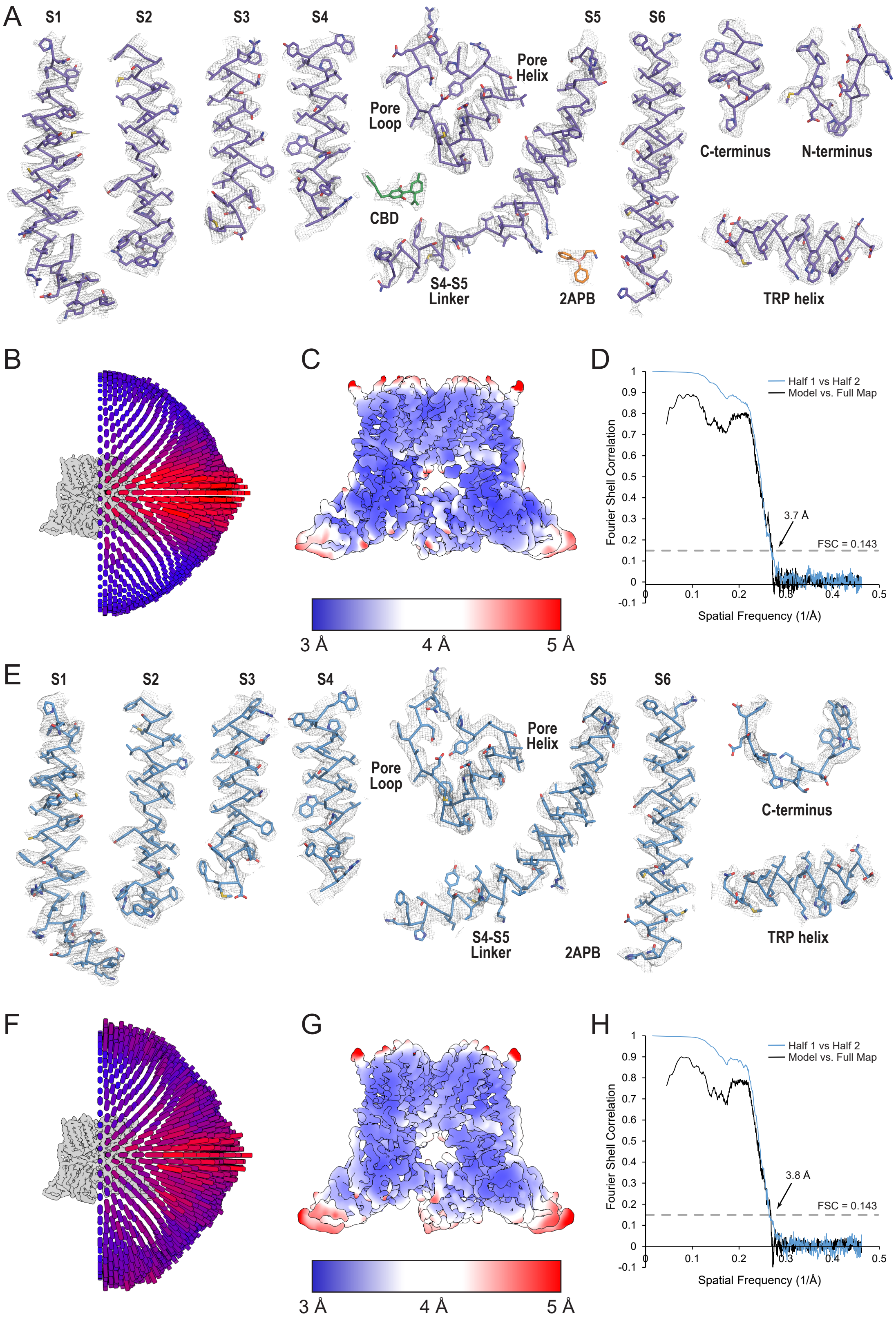

Supplementary Figure 8. Analysis of TRPV2$_{\text{2APB_CBD}}$ cryo-EM data quality. (A) Representative densities of the TRPV2$_{\text{2APB_CBD_AC}}$ cryo-EM map. Model is depicted as a ribbon with sidechains as sticks; all densities are contoured at $\sigma = 4.5$. (B) Angular distribution of particles in the final TRPV2$_{\text{2APB_CBD_AC}}$ C4 refinement. (C) Local resolution of the TRPV2$_{\text{2APB_CBD_AC}}$ map. (D) FSC curves of the final TRPV2$_{\text{2APB_CBD_AC}}$ map and model validation. (E) Representative densities of the TRPV2$_{\text{2APB_CBD_IAC}}$ cryo-EM map. Model is depicted as a ribbon with sidechains as sticks; all densities are contoured at $\sigma = 4.5$. (F) Angular distribution of particles in the final TRPV2$_{\text{2APB_CBD_IAC}}$ C4 refinement. (G) Local resolution of the TRPV2$_{\text{2APB_CBD_IAC}}$ map. (H) FSC curves of the final TRPV2$_{\text{2APB_CB_IAC}}$ map and model validation.

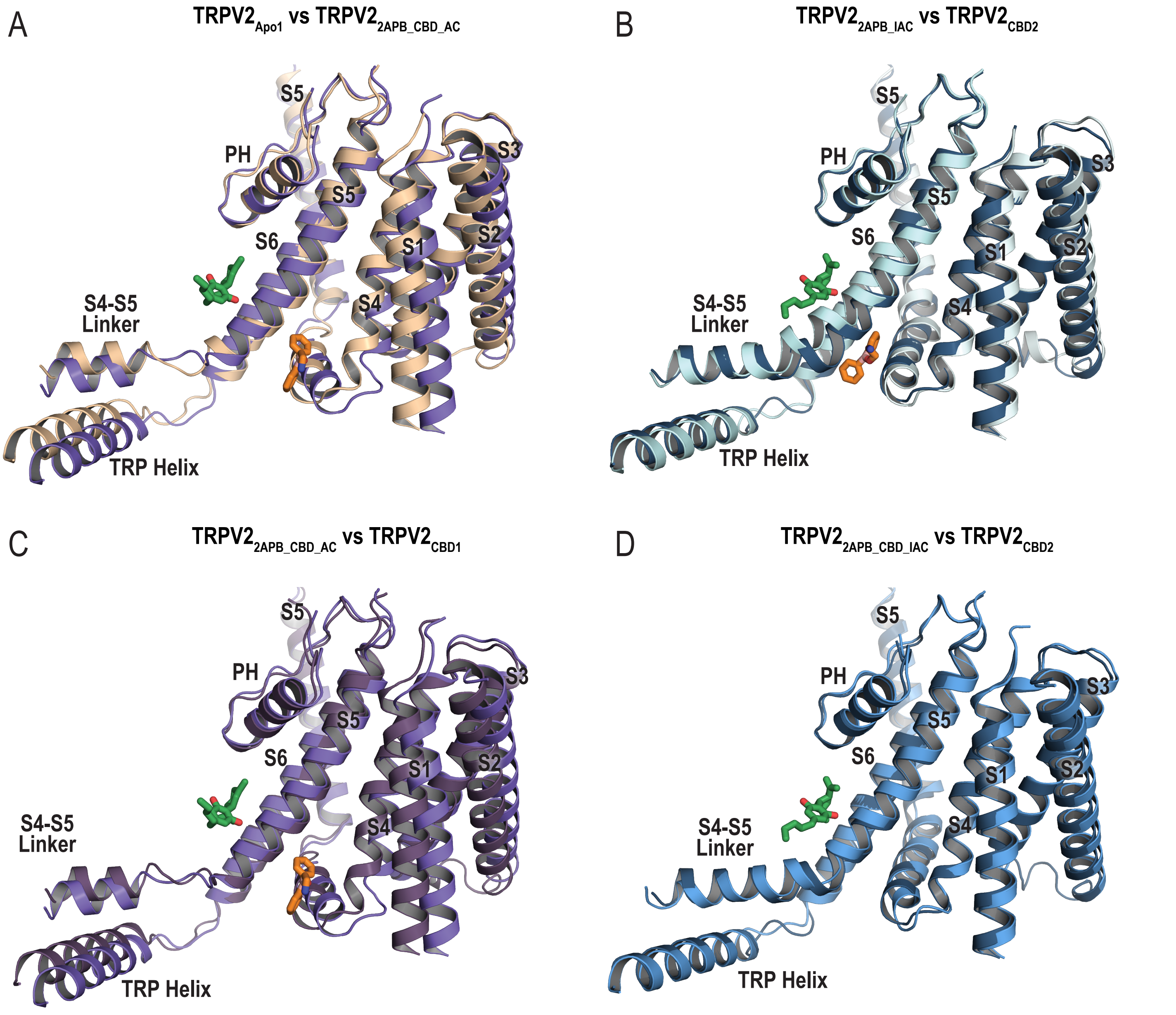

Supplementary Figure 9. Effect of simultaneous binding of 2-APB and CBD to TRPV2 on the channel's conformation. Cartoon representations comparing (A) TRPV2$_{2APB_CBD_AC}$ (medium purple) and TRPV2$_{Apo1}$ (wheat), the drugs shown are from TRPV2$_{2APB_CBD_AC}$; (B) TRPV2$_{2APB_IAC}$ (pale cyan) and TRPV2$_{CBD2}$ (dark blue), the drugs shown are from both structures; (C) TRPV2$_{2APB_CBD_AC}$ (medium purple) and TRPV2$_{CBD1}$ (dark purple), the drugs shown are only from TRPV2$_{2APB_CBD_AC}$; (D) TRPV2$_{2APB_CBD_IAC}$ (medium blue) and TRPV2$_{CBD2}$ (dark blue), the drug shown is from TRPV2$_{CBD2}$. Models were aligned to S6.

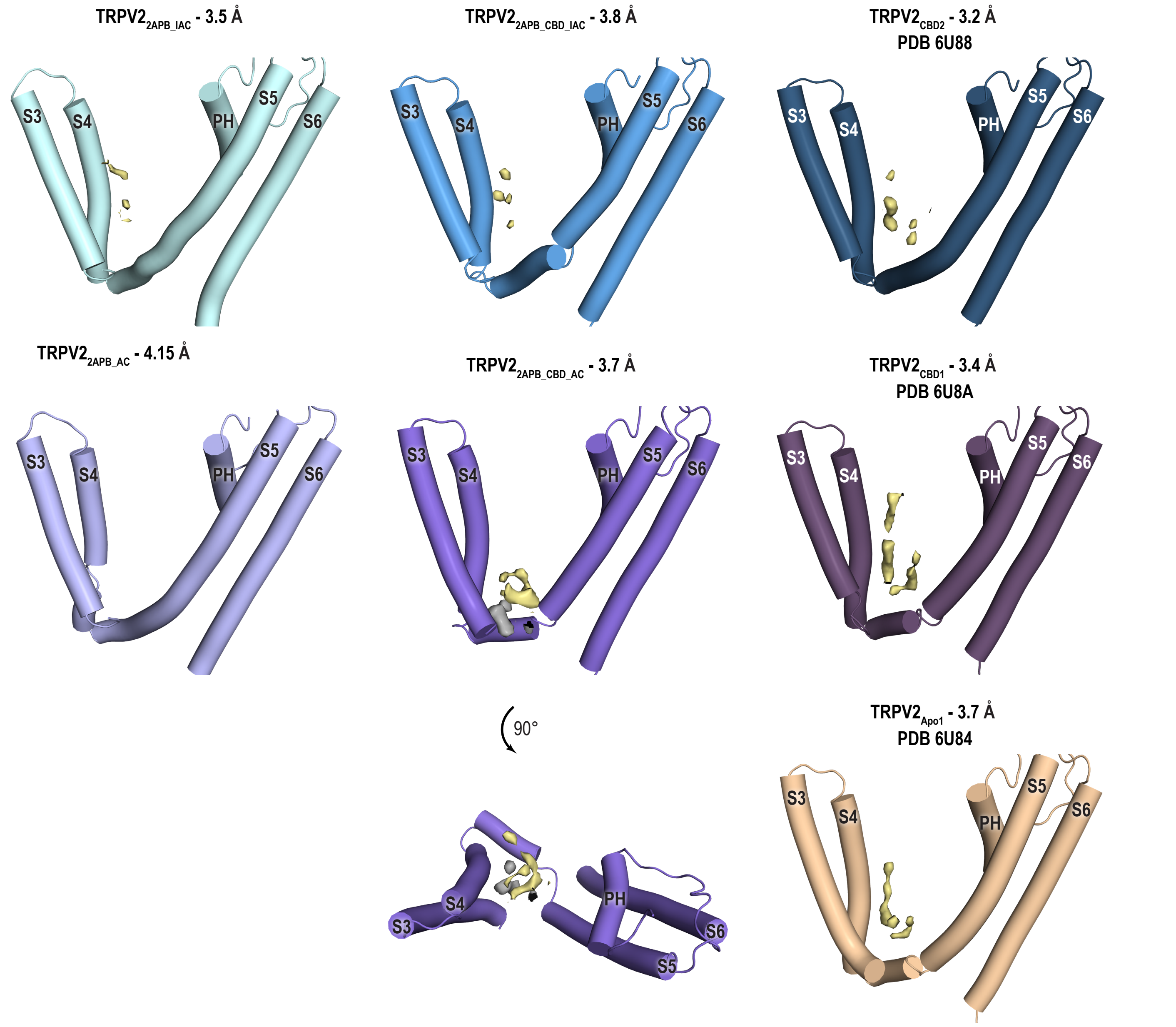

Supplementary Figure 10. Comparison of non-protein densities observed in the vanilloid pocket of various TRPV2 structures. Models are depicted as cylinders. Density maps in the vanilloid pockets are shown at $\sigma = 5$. In TRPV2$_{2APB_AC}$ the lipid cannot be resolved, but it is unclear if this is due to the absence of lipid or to the quality of the cryo-EM map.

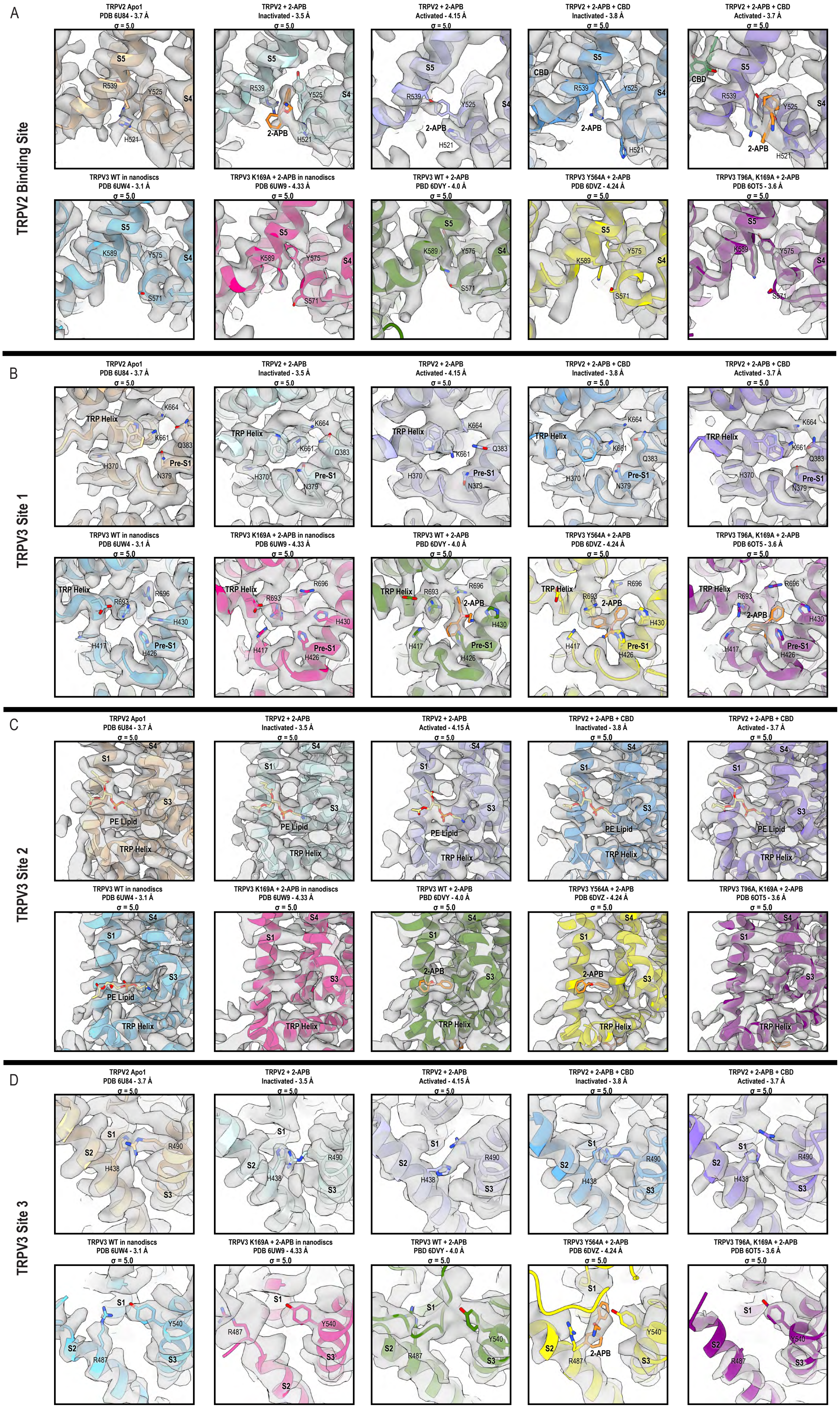
A
TRPV2 Binding Site
TRPV2 Apo1 PDB 6U84 - 3.7 Å σ = 5.0
TRPV2 + 2-APB Inactivated - 3.5 Å σ = 5.0
TRPV2 + 2-APB Activated - 4.15 Å σ = 5.0
TRPV2 + 2-APB + CBD Inactivated - 3.8 Å σ = 5.0
TRPV2 + 2-APB + CBD Activated - 3.7 Å σ = 5.0
TRPV3 WT in nanodiscs PDB 6UW4 - 3.1 Å σ = 5.0
TRPV3 K169A + 2-APB in nanodiscs PDB 6UW9 - 4.33 Å σ = 5.0
TRPV3 WT + 2-APB PBD 6DVY - 4.0 Å σ = 5.0
TRPV3 Y564A + 2-APB PDB 6DVZ - 4.24 Å σ = 5.0
TRPV3 T96A, K169A + 2-APB PDB 6OT5 - 3.6 Å σ = 5.0
S5
S4
R539
Y525
H521
2-APB
CBD
K589
Y575
S571
B
TRPV3 Site 1
TRP Helix
K664
K661
Q383
Pre-S1
H370
N379
R693
R696
H430
H417
H426
C
TRPV3 Site 2
S1
S3
PE Lipid
D
TRPV3 Site 3
S2
H438
R490
R487
Y540

Supplementary Figure 11. Comparison of 2-APB binding sites in TRPV2-TRPV3 structures and cryo-EM maps. (A) TRPV2 2-APB binding site found in the current study is empty in the published TRPV3 structures; (B) Consensus TRPV3 2-APB-binding site near His426 (Site 1) is empty in TRPV2 structures; (C) TRPV3 2-APB-binding site in the VSLD pocket (Site 2) is occupied by a lipid, modeled as PE here, in TRPV2 structures in presence and absence of 2-APB; (D) TRPV3 2-APB-binding site near Tyr540 (Site 3) is occupied by a sidechain in TRPV2 structures. All models are depicted as cartoons with selected sidechains and drugs shown as sticks. All density maps are shown at σ = 5. Reference PDB numbers are shown in the figure.

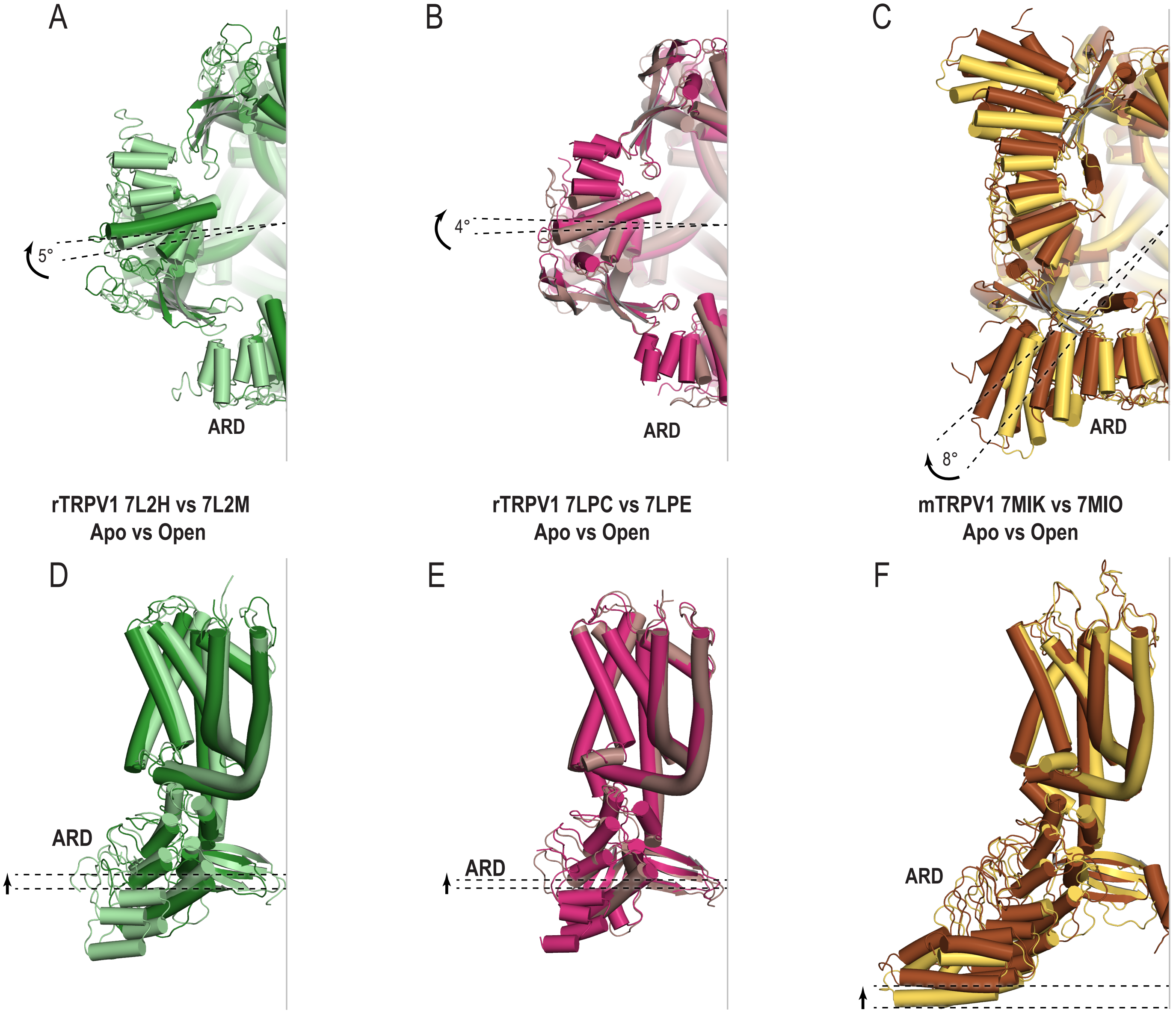

Supplementary Figure 12. Global channel movements observed in the gating cycle of TRPV1 and TRPV3 are similar to those of TRPV2. Bottom views show clockwise rotation of ARDs upon activation in (A) rat TRPV1 DkTx + RTX Open (7L2M, dark green) compared to rat TRPV1 Apo (7L2H, light green); (B) rat TRPV1 Capsaicin, 48ºC Open (7LPE, pink) compared to rat TRPV1 48ºC Closed/Apo (7LPC, light brown); (C) mouse TRPV3 42ºC Open (7MIO, dark brown) compared to mouse TRPV3 42ºC Closed/Apo (7MIK, yellow). Side views show an upward movement of ARDs upon activation in (D) rat TRPV1 DkTx + RTX Open (7L2M, dark green) compared to rat TRPV1 Apo (7L2H, light green); (E) rat TRPV1 Capsaicin, 48ºC Open (7LPE, pink) compared to rat TRPV1 48ºC Closed/Apo (7LPC, light brown); (F) mouse TRPV3 42ºC Open (7MIO, dark brown) compared to mouse TRPV3 42ºC Closed/Apo (7MIK, yellow). All models are depicted as cylinders.

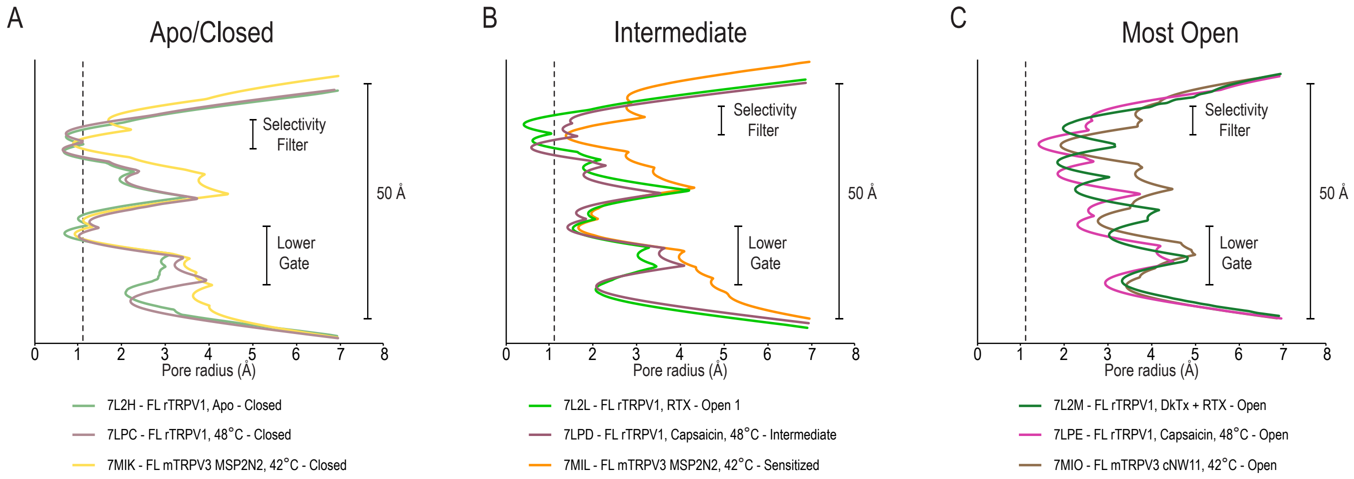

Supplementary Figure 13. Pore profiles of TRPV1 and TRPV3 structures. (A) Closed/Apo states of TRPV1 and TRPV3; (B) Intermediate activated states of TRPV1 and TRPV3; (C) Most open states of TRPV1 and TRPV3. The radius of a dehydrated calcium ion is marked by a dotted grey line at 1.1 Å. Reference PDB numbers are shown in the figure.

Supplementary Table 1. Comparison of full-length TRPV1-TRPV3 structures. All structures taken for comparison were obtained in nanodiscs and refined with C4 symmetry. Most structures are wild type proteins except human TRPV3 structures that carry a K169A mutation which disrupts the cytoplasmic regulatory switch. Radius of the pore at the selectivity filter and at the lower gate was measured by Hole, values above 1.1 Å (the radius of a dehydrated calcium ion) are highlighted in green.

Structures	Mutations	Activation	SF, Å	LG, Å	S6	C-term	N-term	Ref	PDB #
				**Rat TRPV2**					
TRPV2$_{\text{Apo_1}}$	WT	-	0.2	0.3	α	helix	loop	21	6U84
TRPV2$_{\text{Apo_2}}$	WT	-	2.0	0.7	α	helix	loop	21	6U86
TRPV2$_{\text{2APB_AC}}$	WT	2-APB	2.0	1.9	α	helix	loop	this study	7N0N
TRPV2$_{\text{2APB_IAC}}$	WT	2-APB	0.6	0.9	α	loop	-	this study	7N0M
TRPV2$_{\text{2APB_CBD_AC}}$	WT	2-APB, CBD	0.8	1.3	α	helix	loop	this study	7T37
TRPV2$_{\text{2APB_CBD_IAC}}$	WT	2-APB, CBD	0.6	0.7	α	loop	-	this study	7T38
TRPV2$_{\text{CBD_1}}$	WT	CBD	0.6	0.5	α	helix	loop	21	6U8A
TRPV2$_{\text{CBD_2}}$	WT	CBD	0.5	0.8	α	loop	-	21	6U88
				**Rat TRPV1**					
TRPV1$_{\text{Apo}}$	WT	-	0.7	0.7	π	-	-	45	7L2H
TRPV1$_{\text{RTX}}$	WT	RTX	0.4	1.6	π	-	-	45	7L2L
TRPV1$_{\text{RTX DkTX}}$	WT	RTX, DkTX	1.9	3.0	π	-	-	45	7L2M
TRPV1$_{48°}$	WT	48°	0.7	1.0	π	-	-	44	7LPC
TRPV1$_{\text{48° Capsaicin Intermediate}}$	WT	48°, Capsaicin	0.6	1.4	π	-	-	44	7LPD
TRPV1$_{\text{48° Capsaicin Open}}$	WT	48°, Capsaicin	1.4	2.3	π	-	-	44	7LPE
				**Human TRPV3**					
TRPV3$_{\text{Apo}}$	WT	-	0.6	0.8	π	loop	-	24	6UW4
TRPV3$_{\text{K169A}}$	K169A	-	1.8	2.7	π	helix	-	24	6UW6
TRPV3$_{\text{K169A_2-APB_3 min}}$	K169A	2-APB	1.6	1.9	π	helix	-	24	6UW8
TRPV3$_{\text{K169A_2-APB}}$	K169A	2-APB	1.4	0.6	α	helix	-	24	6UW9
				**Mouse TRPV3**					
TRPV3$_{\text{MSP2N2_42°_Closed}}$	WT	42°	0.9	1.0	π	loop	-	43	7MIK
TRPV3$_{\text{MSP2N2_42°_Sensitized}}$	WT	42°	1.4	1.7	π	helix	loop	43	7MIL
TRPV3$_{\text{CNW11_42°_Open}}$	WT	42°	1.9	2.8	π	helix	loop	43	7MIO

Supplementary Note 1. Rosetta scripts for refinement of TRPV2 structure.

Rosetta command lines:

```
/home/tigerous/Rosetta/main/source/bin/rosetta_scripts.linuxgccrelease \
  -database /home/tigerous/Rosetta/main/database/ \
  -in::file::s $pdb \
  -parser::protocol $xml \
  -ignore_unrecognized_res \
  -edensity::mapreso 3.6 \
  -default_max_cycles 200 \
  -relax:constrain_relax_to_start_coords \
  -edensity::cryoem_scatterers \
  -use_input_sc \
  -beta \
  -missing_density_to_jump \
  -out::prefix EM-relax-density- \
  -crystal_refine \
  -nstruct 5
```

Rosetta XML scripts:

```
<ROSETTASCRIPTS>
   <SCOREFXNS>
      <ScoreFunction name="beta" weights="beta_cart"/>
      <ScoreFunction name="dens" weights="beta_cart">
         <Reweight scoretype="elec_dens_fast" weight="35.0"/>
         <Set
scale_sc_dens_byres="R:0.76,K:0.76,E:0.76,D:0.76,M:0.76,C:0.81,Q:0.81,H:0.81,N:0.81,T:0.8
1,S:0.81,Y:0.88,W:0.88,A:0.88,F:0.88,P:0.88,I:0.88,L:0.88,V:0.88"/>
      </ScoreFunction>
   </SCOREFXNS>

   <MOVERS>
       <SetupForDensityScoring name="setupdens"/>
       <LoadDensityMap name="loaddens" mapfile="../
cryosparc_P9_J203_004_volume_map_sharp_35A_107apix.mrc "/>
       <FastRelax name="relaxcart" ramp_down_constraints="false" scorefxn="dens"
repeats="2" cartesian="1"/>
   </MOVERS>

   <PROTOCOLS>
      <Add mover="setupdens"/>
      <Add mover="loaddens"/>
      <Add mover="relaxcart"/>
   </PROTOCOLS>
   <OUTPUT scorefxn="beta"/>

</ROSETTASCRIPTS>
```

Supplementary Note 2. Rosetta scripts for docking of 2-APB.

Rosetta command lines:

```
/home/tigerous/Rosetta/main/source/bin/rosetta_scripts.linuxgccrelease \
    -in:path:database /home/tigerous/Rosetta/main/database \
    -in:file:fullatom \
    -in:file:native $native \
    -in:file:s $pdb \
    -parser:protocol $xml \
    -in:file:extra_res_fa $params \
    -optimization:default_max_cycles 200 \
    -nstruct 20 \
    -use_input_sc \
    -ex1 \
    -ex2 \
    -ex3 \
    -extrachi_cutoff 3 \
    -flip_HNQ \
    -restore_pre_talaris_2013_behavior \
    -overwrite
```

Rosetta XML scripts:

```
<ROSETTASCRIPTS>
    <SCOREFXNS>
        <ScoreFunction name="ligand_soft_rep" weights="ligand_soft_rep">
            <Reweight scoretype="fa_elec" weight="0.42"/>
            <Reweight scoretype="hbond_bb_sc" weight="1.3"/>
            <Reweight scoretype="hbond_sc" weight="1.3"/>
            <Reweight scoretype="rama" weight="0.2"/>
        </ScoreFunction>

        <ScoreFunction name="ligand" weights="ligand">
            <Reweight scoretype="fa_intra_rep" weight="0.004"/>
            <Reweight scoretype="fa_elec" weight="0.42"/>
            <Reweight scoretype="hbond_bb_sc" weight="1.3"/>
            <Reweight scoretype="hbond_sc" weight="1.3"/>
            <Reweight scoretype="rama" weight="0.2"/>
        </ScoreFunction>
    </SCOREFXNS>
    <LIGAND_AREAS>
        <LigandArea name="docking_sidechain" chain="X" cutoff="6.0" add_nbr_radius="true"
all_atom_mode="true" minimize_ligand="10"/>
        <LigandArea name="final_sidechain" chain="X" cutoff="6.0" add_nbr_radius="true"
all_atom_mode="true"/>
        <LigandArea name="final_backbone" chain="X" cutoff="7.0" add_nbr_radius="false"
all_atom_mode="true" Calpha_restraints="0.1"/>
    </LIGAND_AREAS>

    <INTERFACE_BUILDERS>
```

```
        <InterfaceBuilder name="side_chain_for_docking"
ligand_areas="docking_sidechain"/>
        <InterfaceBuilder name="side_chain_for_final" ligand_areas="final_sidechain"/>
        <InterfaceBuilder name="backbone" ligand_areas="final_backbone"
extension_window="3"/>
    </INTERFACE_BUILDERS>

    <MOVEMAP_BUILDERS>
        <MoveMapBuilder name="docking" sc_interface="side_chain_for_docking"
minimize_water="false"/>
        <MoveMapBuilder name="final" sc_interface="side_chain_for_final"
bb_interface="backbone" minimize_water="false"/>
    </MOVEMAP_BUILDERS>

    <SCORINGGRIDS ligand_chain="X" width="20">
        <ClassicGrid grid_name="vdw" weight="1.0"/>
    </SCORINGGRIDS>

    <MOVERS>
        <Transform name="transform" chain="X" box_size="3.0" move_distance="0.1"
angle="5" cycles="1000" repeats="1" temperature="5" initial_perturb="0.5"/>
        <HighResDocker name="high_res_docker" cycles="6" repack_every_Nth="3"
scorefxn="ligand_soft_rep" movemap_builder="docking"/>
        <FinalMinimizer name="final" scorefxn="ligand" movemap_builder="final"/>
        <InterfaceScoreCalculator name="add_scores" chains="X" scorefxn="ligand"
compute_grid_scores="0"/>

        <ParsedProtocol name="low_res_dock">
            <Add mover_name="transform"/>
        </ParsedProtocol>

        <ParsedProtocol name="high_res_dock">
            <Add mover_name="high_res_docker"/>
            <Add mover_name="final"/>
        </ParsedProtocol>

        <ParsedProtocol name="reporting">
            <Add mover_name="add_scores"/>
            Add mover_name="system_name"
        </ParsedProtocol>
    </MOVERS>

    <PROTOCOLS>
        <Add mover_name="low_res_dock"/>
        <Add mover_name="high_res_dock"/>
        <Add mover_name="reporting"/>
    </PROTOCOLS>

</ROSETTASCRIPTS>
```
