# Supplementary material for: Structural insights into TRPV2 activation by small molecules
Source: Nat Commun. 2022 Apr 28;13:2334. doi: 10.1038/s41467-022-30083-3 (PMC9051106; doi:10.1038/s41467-022-30083-3)
Supplement: Supplementary file 2 — Description of Additional Supplementary Files [file 41467_2022_30083_MOESM2_ESM.pdf]

Description of Additional Supplementary Files

File name: Supplementary Movie 1

Description: The C4 mode of movement for 2-APBbound TRPV2 from CryoSPARC processing showing substantial conformational changes and partial opening of the channel.

File name: Supplementary Movie 2

Description: Transitions between structures of $TRPV2_{Apo1}$ (wheat), $TRPV2_{2APB\_AC}$ (light blue), and $TRPV2_{2APB\_IAC}$ (light cyan).
