# Supplementary material for: Structural insights into TRPV2 activation by small molecules
Source: Nat Commun. 2022 Apr 28;13:2334. doi: 10.1038/s41467-022-30083-3 (PMC9051106; doi:10.1038/s41467-022-30083-3)
Supplement: Supplementary file 5 — Reporting Summary [file 41467_2022_30083_MOESM5_ESM.pdf]

nature research

Corresponding author(s): Vera Moiseenkova-Bell
Last updated by author(s): Apr 1, 2022

Reporting Summary

Nature Research wishes to improve the reproducibility of the work that we publish. This form provides structure for consistency and transparency in reporting. For further information on Nature Research policies, see our Editorial Policies and the Editorial Policy Checklist.

Statistics

For all statistical analyses, confirm that the following items are present in the figure legend, table legend, main text, or Methods section.

n/a	Confirmed	
☐	☒	The exact sample size (*n*) for each experimental group/condition, given as a discrete number and unit of measurement
☐	☒	A statement on whether measurements were taken from distinct samples or whether the same sample was measured repeatedly
☐	☒	The statistical test(s) used AND whether they are one- or two-sided<br>*Only common tests should be described solely by name; describe more complex techniques in the Methods section.*
☒	☐	A description of all covariates tested
☐	☒	A description of any assumptions or corrections, such as tests of normality and adjustment for multiple comparisons
☐	☒	A full description of the statistical parameters including central tendency (e.g. means) or other basic estimates (e.g. regression coefficient) AND variation (e.g. standard deviation) or associated estimates of uncertainty (e.g. confidence intervals)
☐	☒	For null hypothesis testing, the test statistic (e.g. *F*, *t*, *r*) with confidence intervals, effect sizes, degrees of freedom and *P* value noted<br>*Give P values as exact values whenever suitable.*
☒	☐	For Bayesian analysis, information on the choice of priors and Markov chain Monte Carlo settings
☒	☐	For hierarchical and complex designs, identification of the appropriate level for tests and full reporting of outcomes
☒	☐	Estimates of effect sizes (e.g. Cohen's *d*, Pearson's *r*), indicating how they were calculated

Our web collection on statistics for biologists contains articles on many of the points above.

Software and code

Policy information about availability of computer code

Data collection	EPU Software, Patchmaster v2x53
Data analysis	Clustal Omega, Aline, Relion 3.1, cryoSPARC v3.2, Coot 0.8.9.3, Chimera software package, Chimera X software package, PHENIX software package, Patchmaster/Fitmaster software, Fitmaster v2x90.5, Origin 8.5.1, OpenEye Scientific Software

For manuscripts utilizing custom algorithms or software that are central to the research but not yet described in published literature, software must be made available to editors and reviewers. We strongly encourage code deposition in a community repository (e.g. GitHub). See the Nature Research guidelines for submitting code & software for further information.

Data

Policy information about availability of data

All manuscripts must include a data availability statement. This statement should provide the following information, where applicable:
- Accession codes, unique identifiers, or web links for publicly available datasets
- A list of figures that have associated raw data
- A description of any restrictions on data availability

The atomic coordinates and cryo-EM density maps generated in this study have been deposited in the Protein Data Bank and Electron Microscopy Data Bank under the accession codes TRPV22APB_AC (PDB 7N0N [http://doi.org/10.2210/pdb7N0N/pdb] and EMD-24110 [https://www.emdataresource.org/EMD-24110]), TRPV22APB_IAC (PDB 7N0M [http://doi.org/10.2210/pdb7N0M/pdb] and EMD-24109 [https://www.emdataresource.org/EMD-24109]), TRPV22APB_CBD_AC (PDB 7T37 [http://doi.org/10.2210/pdb7T37/pdb] and EMD-25650 [https://www.emdataresource.org/EMD-25650]), TRPV22APB_CBD_IAC (PDB 7T38 [http://doi.org/10.2210/pdb7T38/pdb] and EMD-25651 [https://www.emdataresource.org/EMD-25651]). The atomic coordinates and cryo-EM density maps of previously solved TRPV2 structures used in this study are available in the Protein Data Bank and Electron Microscopy Data Bank under the accession codes TRPV2Apo1 (PDB 6U84 [http://doi.org/10.2210/pdb6U84/pdb] and EMD-20677 [https://www.emdataresource.org/EMD-20677]), TRPV2CBD1 (PDB 6U8A [http://doi.org/10.2210/

pdb6U8A/pdb and EMD-20686 [https://www.emdataresource.org/EMD-20686]), TRPV2CBD2 (PDB 6U88 [http://doi.org/10.2210/pdb6U88/pdb] and EMD-20682 [https://www.emdataresource.org/EMD-20682]), TRPV2 ARD (PDB 2ETA [http://doi.org/10.2210/pdb2ETA/pdb]).
All electrophysiological patch clamp data supporting the conclusions drawn in this study are available within the original article and/or in the supplement. Additional information such as raw data of patch clamp recordings are available from the corresponding author upon reasonable request. Plasmids of the rTPRV2-mutant constructs investigated and presented in this study are available from the corresponding author upon request.

Field-specific reporting

Please select the one below that is the best fit for your research. If you are not sure, read the appropriate sections before making your selection.

☒ Life sciences ☐ Behavioural & social sciences ☐ Ecological, evolutionary & environmental sciences

For a reference copy of the document with all sections, see nature.com/documents/nr-reporting-summary-flat.pdf

Life sciences study design

All studies must disclose on these points even when the disclosure is negative.

Sample size

Variance and effect size were not known before this study, thus a valid sample size predetermination was not performed. The properties of mutant rTRPV2-channels constructed by site-directed-mutagenesis according to assumptions drawn from Cryo-EM data, e.g. the effect size was not known. In our hands, experiments for concentration-dependent activation required to obtain EC50-curves usually display little variability between individual cells. 4-10 cells per mutant construct are usually sufficient for valid and conclusive data.

Data exclusions

In whole-cell patch clamp experiments, cell were only included when the baseline remained stable with a low leak current (< 100 pA) throughout the complete experiment containing clear effects for all 2-APB-concentrations intended to be explored. All cells had an initial giga-seal.

Replication

Patch Clamp experiments were conducted on cells from several independent transfections. In order to ensure for close to identical experimental conditions - including identical 2-APB test solutions - the experiments for creating dose-response curves for 2-APB-induced activation were performed within 2-3 days. Control experiments with wildtype rTRPV2 were regularly conducted in order to monitor replication.

Randomization

Randomization is used in cryoSPARC and Relion software when estimating the resolution of the final cryo-EM maps.
For patch clamp experiments, investigated individual cells are randomly chosen by the experimenter from usually >100 transfected cells in the petri dish. Only one cell per dish is investigated. The chosen cells must be attached to the petri dish, look “healthy” with a clear cell membrane and show an intense fluorescence indicating successful transfection.

Blinding

A true blinding was not possible, as the patch clamp experimenters also performed the transfection procedures. At the time of the transfection however, the multiple rTRPV2-mutant constructs were only marked according to an ongoing numbering of mutants created in the laboratory (e.g. M34, M35, M36 etc., M for mutant). The experimenter did not know the exact nature of the mutant at the time of the experiment, this was revealed at the time of data analysis. Furthermore, the experimenters were not informed about underlying the Cyro-EM data before the experiment. Thus, they did not know what was predicted.

Reporting for specific materials, systems and methods

We require information from authors about some types of materials, experimental systems and methods used in many studies. Here, indicate whether each material, system or method listed is relevant to your study. If you are not sure if a list item applies to your research, read the appropriate section before selecting a response.

Materials & experimental systems

n/a	Involved in the study	
☐	☒	Antibodies
☐	☒	Eukaryotic cell lines
☒	☐	Palaeontology and archaeology
☒	☐	Animals and other organisms
☒	☐	Human research participants
☒	☐	Clinical data
☒	☐	Dual use research of concern

Methods

n/a	Involved in the study	
☒	☐	ChIP-seq
☒	☐	Flow cytometry
☒	☐	MRI-based neuroimaging

Antibodies

Antibodies used

We used 1D4 antibodies for protein purification

Validation

Describe the validation of each primary antibody for the species and application, noting any validation statements on the manufacturer’s website, relevant citations, antibody profiles in online databases, or data provided in the manuscript.

Eukaryotic cell lines

Policy information about cell lines

Cell line source(s)	An HEK293T cell line were from ATCC
Authentication	The HEK293T cell line was not authenticated
Mycoplasma contamination	The HEK 293T cells are regularly tested for mycoplasma contamination (negative results). Cells are regularly replaced by fresh batches free form mycoplasma.
Commonly misidentified lines (See ICLAC register)	No commonly misidentified cell lines were used in this study
